# Overcoming the Conductance versus Crossover Trade-off in State-of-the-Art Proton Exchange Fuel-Cell Membranes by Incorporating Atomically Thin Chemical Vapor Deposition Graphene

**DOI:** 10.1021/acs.nanolett.4c05725

**Published:** 2025-01-13

**Authors:** Nicole K. Moehring, Abdul Bashith Mansoor Basha, Pavan Chaturvedi, Thomas Knight, Xiaozong Fan, Peter N. Pintauro, Michael S. H. Boutilier, Kunal Karan, Piran R. Kidambi

**Affiliations:** † Interdisciplinary Graduate Program in Materials Science, Vanderbilt University, Nashville, Tennessee 37235, United States; ‡ Chemical and Biomolecular Engineering Department, Vanderbilt University, Nashville, Tennessee 37212, United States; § Vanderbilt Institute of Nanoscale Science and Engineering, Nashville, Tennessee 37212, United States; ∥ Department of Chemical and Petroleum Engineering, University of Calgary, Calgary, AB T2N 1N4, Canada; ⊥ Department of Chemical and Biochemical Engineering, Western University, London, Ontario N6A 5B9, Canada; # Department of Chemistry, Vanderbilt University, Nashville, Tennessee 37235, United States; 7 Mechanical Engineering Department, Vanderbilt University, Nashville, Tennessee 37212, United States; 8 Walker Department of Mechanical Engineering, The University of Texas at Austin, Austin, Texas 78712-1591, United States

**Keywords:** graphene, crossover reduction, proton exchange membrane (PEM), fuel cell, Nafion, chemical vapor deposition (CVD), conductance vs crossover trade-off, permeance vs selectivity

## Abstract

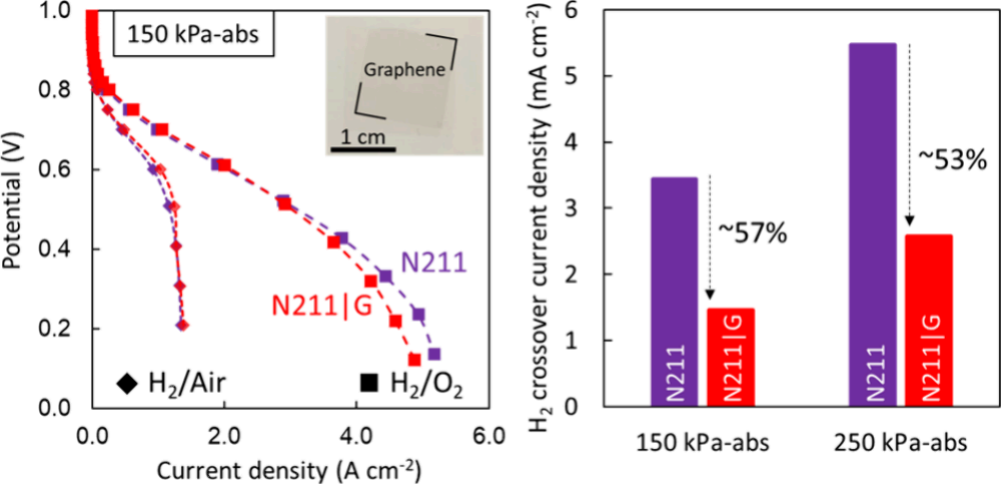

Permeance–selectivity trade-offs are inherent to polymeric membranes. In fuel cells, thinner proton exchange membranes (PEMs) could enable higher proton conductance and increased power density with lower area-specific resistance (ASR), smaller ohmic losses, and lower ionomer cost. However, reducing thickness is accompanied by an increase in undesired species crossover harming performance and long-term efficiency. Here, we show that incorporating atomically thin monolayer graphene synthesized via scalable chemical vapor deposition (CVD) and tunable defect density into PEMs (Nafion, ∼5–25 μm thick) can allow for reduced H_2_ crossover (∼34–78% of Nafion of a similar thickness) while maintaining adequate areal proton conductance for applications (>4 S cm^–2^). In contrast to most prior work using >50 μm symmetric Nafion sandwich structures, we elucidate the interplay of graphene defect density and Nafion proton transport resistance on the performance of Nafion|graphene composite membranes and find high-quality low-defect density CVD graphene (G) supported on Nafion 211 (∼25 μm); i.e., N211|G has a high areal proton conductance (∼6.1 S cm^–2^) and the lowest H_2_ crossover (∼0.7 mA cm^–2^). Fully functional centimeter-scale N211|G fuel-cell membranes demonstrate performance comparable to that of state-of-the-art Nafion N211 at room temperature as well as standard operating conditions (∼80 °C, ∼150–250 kPa-abs) with H_2_/air (power density ∼0.57–0.63 W cm^–2^) and H_2_/O_2_ feed (power density ∼1.4–1.62 W cm^–2^) and markedly reduced H_2_ crossover (∼53–57%).

Proton exchange membranes (PEMs) play an important role in a range of clean power generation^1^ and conversion processes,^2−4^ including hydrogen (H_2_) and methanol fuel cells^5,6^ for transportation and remote/auxiliary power, respectively, redox flow batteries for grid-scale energy storage,^7,8^ electrolysis for H_2_ production, isotope separations,^9^ sustainability,^10,11^ etc.^1−15^ PEM-based H_2_ fuel-cell applications utilize the state-of-the-art ionomer Nafion, a sulfonated fluoropolymer with a fluorocarbon backbone and side chains that are terminated with sulfonic acid that allows for selective proton transport.^8,12−14^ However, Nafion exhibits the classic permeance–selectivity trade-offs endemic to polymeric membranes; i.e., thinner membranes could allow for higher proton conductance and increased stack power density due to lower area-specific resistance and smaller ohmic losses as well as lower ionomer cost, but reduced thickness also results in increased H_2_ crossover, which is detrimental to electrochemical performance and long-term efficiency.^8,12,13^

In this context, atomically thin two-dimensional (2D) materials such as graphene and hexagonal boron nitride (h-BN) present potential for advancing PEMs.^3,5,6,8,13,15−31^ The pristine lattice of graphene and h-BN allows for electric field-driven permeation of protons while hindering transport of even small gas atoms such as He. Leveraging the selective proton transport of 2D materials and integrating them with conventional polymeric PEMs can enable approaches to overcome the permeance–selectivity trade-off in PEMs.^2,4,5,7,8,12,13,16,33−47^

However, PEM applications necessitate large-area synthesis of 2D materials via bottom-up chemical vapor deposition (CVD) processes^4,36,41−43,48−52^ that typically incorporate intrinsic defects into the 2D lattice.^48,49,51,53^ Intrinsic defects could, on one hand, enhance proton transport relative to the pristine lattice,^19,54^ but on the other hand, larger nonselective defects can decrease membrane selectivity due to increased hydrogen crossover.^4,16,37,38,55^ Hence, understanding the complex interplay between intrinsic defects, PEM support resistance, and differences in the transport pathways for H_2_ crossover and proton transport in PEMs is imperative to enable rational design and pathways to integrate atomically thin graphene films with Nafion^4,5,8,12,13,36^ to simultaneously leverage the high proton conductance of selective intrinsic defects as well as reduced H_2_ crossover^4−6,8,36^ due to (i) the impermeability of the pristine 2D lattice and (ii) the low propensity of large defects in 2D materials to precisely overlap with the water channels in the Nafion that typically allow for H_2_ crossover.^4,13,16,22,56^

Here, we systematically study the incorporation of atomically thin monolayer CVD graphene with tunable intrinsic defect density with Nafion of varying thicknesses (∼5–25 μm) for fuel-cell applications. A thin Nafion carrier layer enables facile integration of CVD graphene into ∼5–25 μm PEMs, in contrast to most prior work with symmetric Nafion sandwich structures (>50 μm),^4−6,8,36^ enabling systematic progress toward realistic operational conditions. Using a combination of systematic experiments and detailed resistance modeling, we elucidate the critical trade-offs between the complex interplay of graphene defect density and Nafion support transport resistance and its influence on H_2_ crossover as well as the areal proton conductance of the resulting graphene|Nafion composite membranes. Our results show that the incorporation of monolayer CVD graphene can allow for ∼34–78% reduction in H_2_ crossover compared to that seen with bare Nafion (of a similar thickness), while maintaining an adequate proton conductance of >4 S cm^–2^ for practical applications. Industry standard state-of-the-art Nafion N211 (∼25 μm) interfaced with high-quality (low-defect density) CVD graphene, i.e., N211|G, shows high areal proton conductance (∼6.1 S cm^–2^) as well as the lowest H_2_ crossover (∼0.7 mA cm^–2^) at room temperature under 100% humidity, thereby allowing the conductance–crossover trade-off to be overcome. We demonstrate fully functional centimeter-scale N211|G fuel-cell membranes with significantly reduced H_2_ crossover (∼53–57%) and fuel-cell performance comparable to that of state-of-the-art Nafion N211 with H_2_/air and H_2_/O_2_ feed at room temperature and under standard operating conditions (∼80 °C, ∼150–250 kPa-abs), maintaining power densities of ∼0.57–0.63 W cm^–2^ (H_2_/air) and ∼1.4–1.62 W cm^–2^ (H_2_/O_2_).

## Interfacing Atomically Thin Monolayer CVD Graphene with Nafion for PEMs

**Figure 1:**
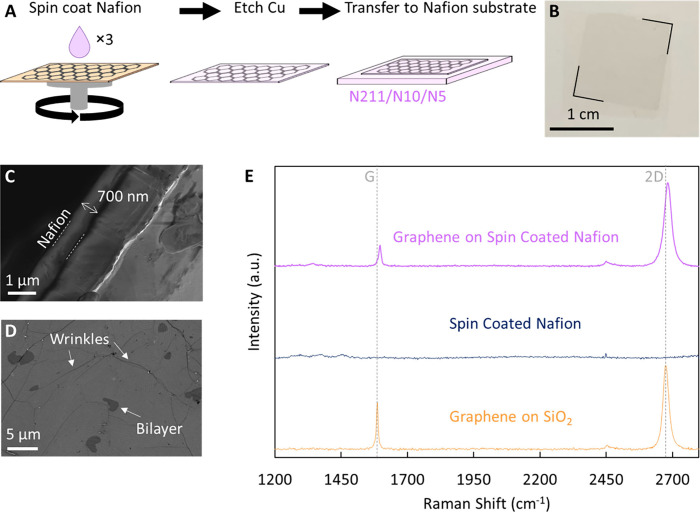
Interfacing graphene with Nafion 211 and PTFE-reinforced Nafion thin films. (A) Schematic of the graphene transfer process. A thin Nafion layer (∼700 nm thick) is spin-coated onto CVD graphene on Cu foil, the Cu etched, and the Nafion–graphene stack scooped onto the desired Nafion support (N211, ∼25 μm thick; N10, ∼10 μm thick; and N5, ∼5 μm thick). (B) Optical image of centimeter-scale graphene transferred to Nafion 211 (black lines are guides for the eye indicating graphene edges). (C) SEM cross-section image of the spin-coated Nafion layer (dotted white lines are just a guide for the eye) on graphene on Cu foil. (D) SEM image of graphene on the spin-coated Nafion film showing wrinkles and minor bilayer patches characteristic of CVD graphene. The absence of ruptures or large/macroscopic damage to graphene suggests high-quality transfer. (E) Raman spectrum for graphene transferred on a 300 nm SiO_2_/Si wafer (orange) showing the characteristic 2D (∼2700 cm^–1^) and G (∼1600 cm^–1^) peaks as well as the absence of a D (∼1350 cm^–1^) peak, indicating the high quality of the as-synthesized CVD graphene. The G and 2D peaks observed in the Raman spectrum for graphene on spin-coated Nafion (purple) compared to the control spin-coated Nafion (blue) without peaks indicate successful transfer. A shift to higher wavenumbers for the G and 2D peaks for graphene on spin-coated Nafion could originate from doping and/or strain of the graphene lattice.^59,60^

**Figure 2:**
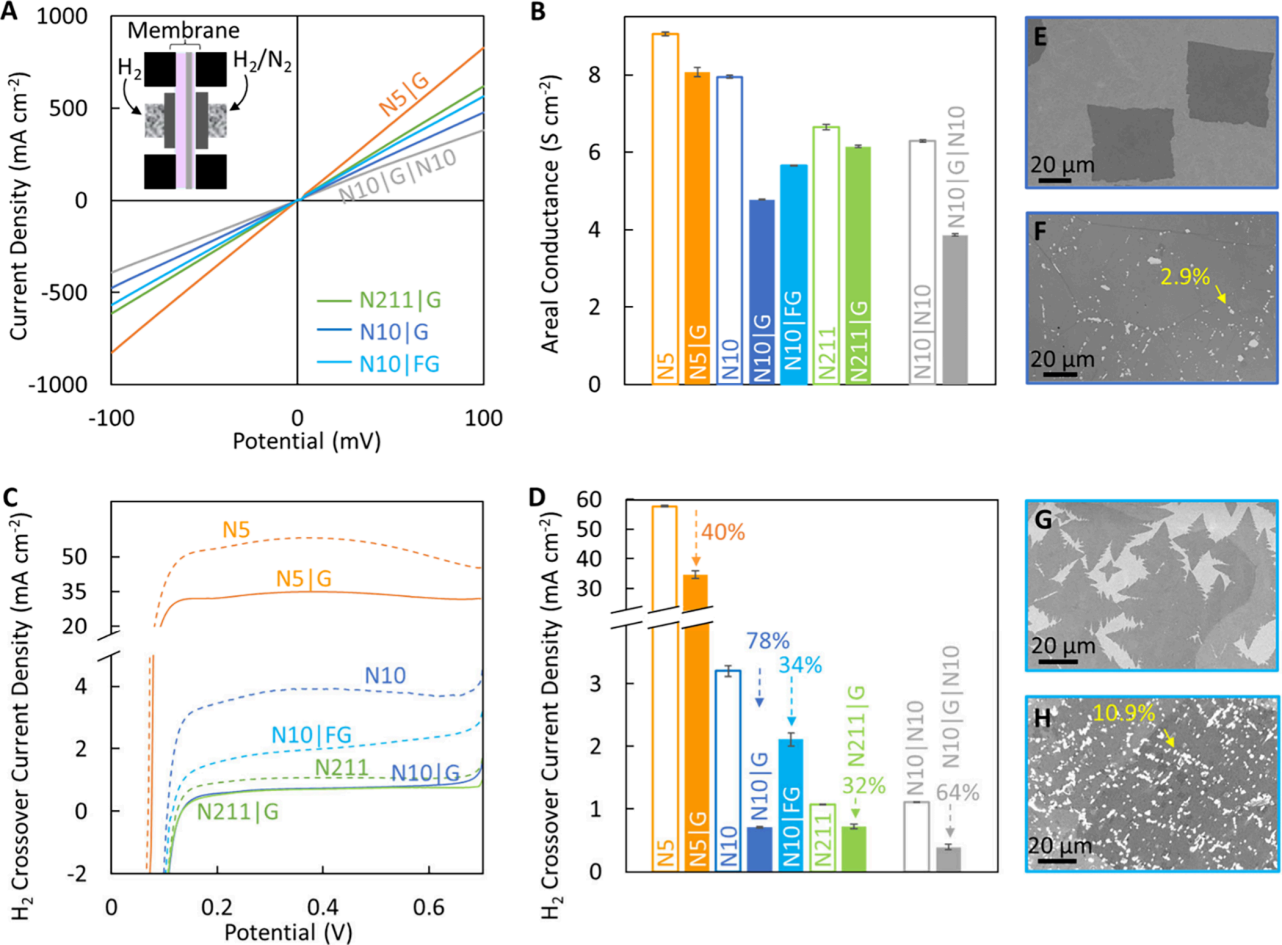
Transport through CVD graphene interfaced with different thicknesses of Nafion (N5, 5 μm; N10, 10 μm; and N211, 25 μm) in a custom-built cell at room temperature. (A) Proton transport is characterized by supplying humidified H_2_ to either side of the membrane (see the inset; active area ∼0.32 cm^2^) and measuring the current at the applied potential for graphene (G) on N5 (N5|G), N10 (N10|G), and N211 (N211|G) as well G in an N10 sandwich (N10|G|N10) and fast graphene (FG) on N10 (N10|FG). (B) Areal conductance extracted from panel A (inverse of slope of the *I–V* plot) for Nafion|graphene composite membranes (filled bars) and corresponding controls (unfilled bars, *I*−*V* curves not shown in A). Error bars represent one standard deviation. (C) H_2_ crossover curves for graphene membranes and respective controls measured with humidified H_2_ on one side and humidified N_2_ on the other.^68^ (D) H_2_ crossover current density extracted from the crossover curves in panel C at 400 mV (as per the DOE standard). Downward-pointing arrows with numbers on top indicate the percent reduction in the level of H_2_ crossover upon the addition of graphene compared to the respective controls. (E) SEM image of graphene (G) domains prior to convergence, showing square graphene domains are obtained by controlling kinetics via growth parameters.^4^ (F) SEM image of continuous CVD graphene films on Cu foil after an electrochemical etch test in which etch pits form in Cu underneath defects in graphene. These etch pits are visible as bright white spots (indicated by yellow arrow), and the percentage area of etch pits is indicated in yellow text (∼2.9%). (G) SEM image of graphene domains with dendritic edges obtained by faster growth (FG) with a higher level of CH_4_ to increase the number of intrinsic defects on the lattice.^4^ (H) SEM image of FG after electrochemical etch test showing the higher percent of etch pit area (∼10.9%), consistent with higher proton conductance and higher H_2_ crossover for N10|FG than for N10|G. Ambient pressure and temperature with equal flow of H_2_ and/or N_2_ to either side were used for all experiments.

To integrate graphene with the Nafion substrate (Figure 1A), a layer of Nafion is first spin-coated on graphene grown on Cu foil via CVD. The ∼700 nm thick (Figure 1C) spin-coated Nafion layer serves as a carrier/support layer for graphene during removal of the Cu foil by acid etching and facilitates facile graphene transfer to the target Nafion substrates of choice to form Nafion|G (Nafion|G|700 nm spin-coated Nafion) composite PEMs (Figure 1A,B).^57^ Additionally, the spin-coated Nafion layer also protects the CVD graphene from coming into direct contact with the Pt/C electrodes during fabrication of membrane electrode assemblies (MEAs) (see schematic in inset of Figure 2A).

The efficacy of graphene transfer is evaluated using optical images (Figure 1B) and scanning electron microscopy (SEM) (Figure 1D). Optical images show large areas of uniform contrast, with no visible cracks or tears at the macroscopic scale (Figure 1B). SEM images show features consistent with graphene films such as wrinkles in the film, small multilayers (Figure 1D), and the absence of significant microscale damage (<0.25% area), indicating very high-fidelity transfer.

Raman spectroscopy of graphene transferred to 300 nm SiO_2_/Si wafer (Figure 1E) shows characteristic peaks of monolayer graphene at ∼2700 cm^–1^ (2D) and ∼1580 cm^–1^ (G) with an *I*_2D_/*I*_G_ ratio of ∼1.8, and a negligible D peak at ∼1350 cm^–1^ (*I*_D_/*I*_G_ ratio of ∼0.032) confirms the high quality of the as-synthesized CVD graphene.^57^ Notably, Nafion peaks at ∼1207 cm^–1^ (E_1_ CF_2_ degenerate stretch), ∼1291 cm^–1^ (E_2_ CC degenerate stretch), and ∼1372 cm^–1^ (A_1_ CC symmetric stretch)^58^ are relatively weak but detectable (Figure 1, spin-coated Nafion) and overlap with the D peak region for graphene. Nonetheless, the successful transfer of graphene to spin-coated Nafion is confirmed by the presence of the characteristic 2D and G peaks, and the minor blue-shift is attributed to doping and/or lattice strain due to the flexibility of the Nafion substrate.^59,60^

## Probing the Interplay between Intrinsic Defects in CVD Graphene and the Nafion Support Resistance on Proton Transport and H_2_ Crossover for PEMs

**Figure 3:**
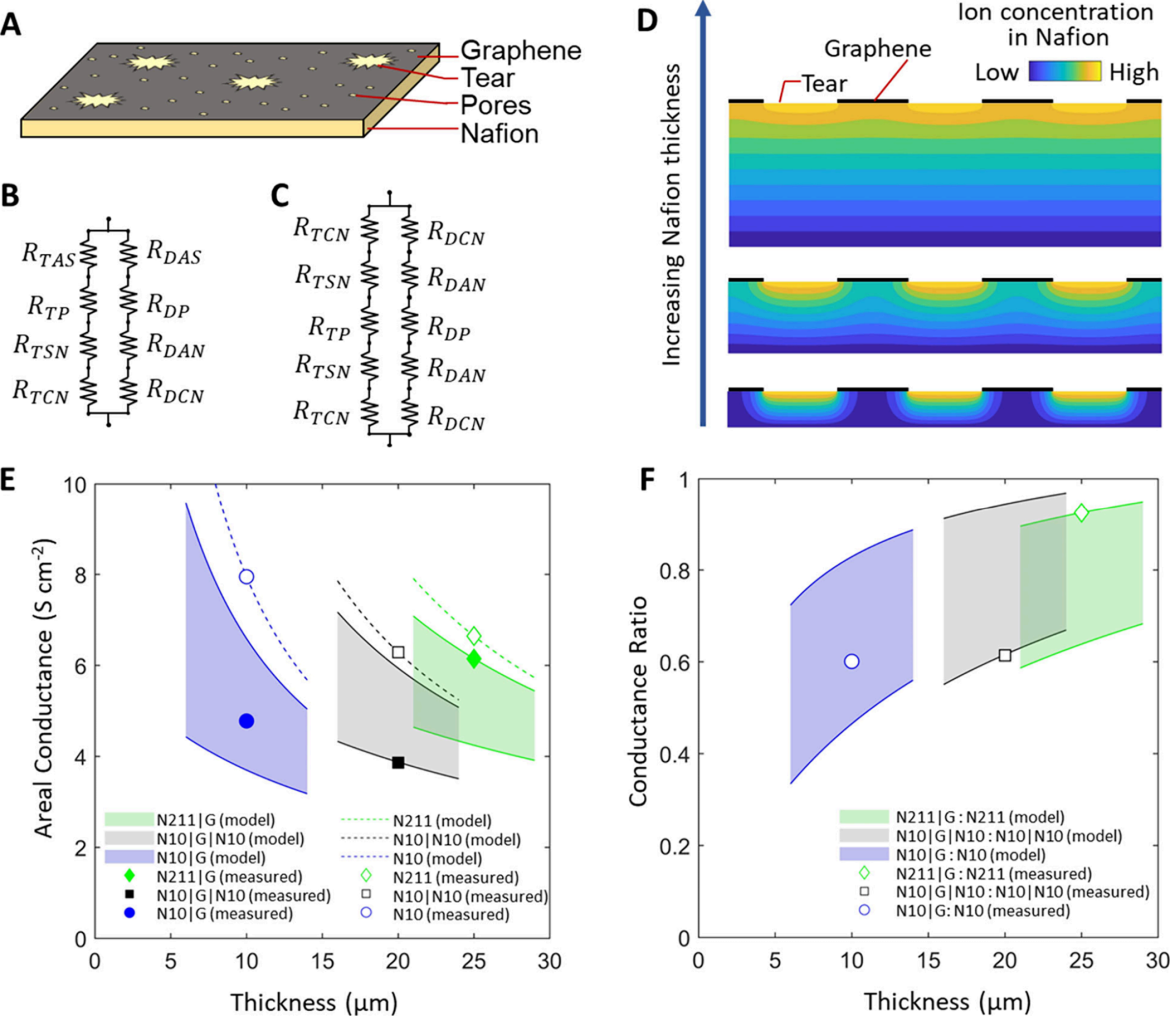
Resistance model for graphene interfaced with Nafion of different thicknesses. (A) Structure of a membrane with one layer of graphene on Nafion showing larger tears and smaller defects in the graphene layer over the Nafion support. Resistance network models for graphene interfaced with (B) one and (C) two Nafion layers. The resistances of a single tear (*R*_TAS_ is the resistance to access the tear, *R*_TP_ the resistance to pass through the tear, *R*_TSN_ the resistance to spread out from the tear, and *R*_TCN_ the resistance to be transported within the Nafion) and a single defect (*R*_DAS_ is the resistance to access the defect, *R*_DP_ the resistance to pass through the defect, *R*_DAN_ the resistance to spread out from the defect, and *R*_DCN_ the resistance to be transported within the Nafion) are shown, though many of each occur in parallel. (D) Illustration of the spreading/constriction resistance effect for different Nafion layer thicknesses, showing the effectively smaller conductance area for membranes that are thin compared to that of tear spacing. (E) Areal conductance and (F) conductance ratio from the model (bands) compared to experimental measurements (symbols, filled for graphene and empty for controls in panel E). Shaded bands show fractional areas occupied by tears ranging from 3.5% to 14%. Conductance measurements for the N211|G membrane correspond to a calculated tear fraction higher than those of the N10|G and N10|G|N10 membranes. Differences in interactions between the nonreinforced (N211) and reinforced (N10) Nafion supports can affect tear formation and have an impact on proton conductance.

To develop an understanding of the interplay between intrinsic defects in graphene, Nafion support resistance, and trade-offs for interfacing atomically thin CVD graphene with Nafion for PEMs, we experimentally study the impact of Nafion support thickness (N211, ∼25 μm thick; N10 ∼10 μm thick; and N5, ∼5 μm thick) on proton transport and H_2_ crossover in conjunction with a resistance model (Figure 3). Notably, N5 and N10 contain an embedded porous PTFE reinforcement structure and showed deformation on a hot press (see Figure S1) compared to conventional N211 (without PTFE reinforcement). Hence, we limit the use of a hot press to adding electrodes for MEA fabrication (see methods) and to make the N10|G|N10 sandwich (∼20 μm) to facilitate a comparison with N211|G (∼25 μm) of similar thickness.

Figure 2A shows representative *I–V* curves acquired at room temperature (RT) via linear sweep voltammetry (LSV) when humidified H_2_ is supplied to both sides of the Nafion|G composite PEMs. The areal proton conductance (siemens per square centimeter) is computed by taking the inverse of the *I–V* slope (Figure 2B). An increase in areal conductance is observed with a decrease in the thickness of Nafion (N211 ∼6.2 S cm^–2^; N10 ∼7.9 S cm^–2^; and N5 ∼9.1 S cm^–2^). Interestingly, the addition of graphene reduces proton conductance by varying amounts for the different Nafion substrates; e.g., for N211|G, ∼7.5% reduction (filled green bar) is observed compared to that of the N211 control (unfilled green bar), consistent with our own prior work as well as other reports in the literature (∼9–11%).^4,18^ However, graphene interfaced with N10 (N10|G, dark blue solid bar) shows ∼39.9% reduction (from ∼7.9 to ∼4.8 S cm^–2^), and these observations are consistent across multiple samples (Figure S2). Finally, N5|G shows ∼10.8% reduction in its proton conductance compared to that of N5 (Figure 2B) despite the differences in processing required (see methods and Figure S1B,C), which is consistent with the relative change in proton conductance observed for N211.

Interestingly, PEMs of similar thickness, i.e., N211 (∼25 μm thick) and N10|N10 sandwich (∼20 μm thick), show similar areal conductance values to protons (N10|N10, 6.3 S cm^–2^; and N211, 6.6 S cm^–2^) despite differences in N10 being reinforced with PTFE while N211 is not. This suggests the PTFE reinforcement has minimal influence on proton transport and also that there is minimal interfacial resistance contribution between the N10 layers (Figure 2B). Notably, the N10|G|N10 sandwich membrane similar in thickness to N211 showed ∼38.5% reduction in the level of proton transport compared to the control sandwich (N10|N10), which is consistent with reduction for N10|G compared to N10.

To understand the origins of the reduction in proton transport for N10|G and in an effort to explore avenues to increase the areal proton conductance, we interfaced graphene grown under different CVD conditions [fast graphene (FG) due to the rate of CVD growth that has been demonstrated to have a higher defect density]^4^ and found the areal proton conductance can indeed be improved from ∼4.8 S cm^–2^ (N10|G) to ∼5.7 S cm^–2^ (N10|FG). The increase in areal conductance is attributed to the presence of an increased number of angstrom-scale defects (confirmed by scanning tunneling microscopy)^4^ in the 2D lattice of FG arising from differences in kinetics during CVD growth.^4^ The kinetic control of the CVD growth process also manifests as differences in graphene domain shapes for G (rectangular, Figure 2E) and FG (dendritic, Figure 2G) prior to convergence to form a continuous film as well as an increase in the density of etch pits formed in the Cu underneath defects in graphene upon subjecting CVD graphene on Cu foil to electrochemical etch tests (Figure 2F,H).^4^ The etch pits appear as bright spots in the SEM image, and the etched area of FG (∼10.9%), which is greater than that of G (∼2.9%), confirms the higher defect density of FG.^16,22^

To gain further insights into the significant reduction in areal proton conductance measured when G is transferred onto N10 compared to N211, we developed an approximate analytical ion transport model. The model builds on prior ion transport resistance modeling for this membrane structure.^4,36^ Without graphene present, the resistance [*R* (ohms)] to ion transport through the Nafion layer is modeled as one-dimensional (1D) conduction. For a Nafion layer of thickness *t*_N_ (meters) and ion conductivity *σ*_N_ (siemens per meter), the transport resistance through one layer of Nafion is
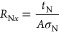
1where *A* (square meters) is the total membrane area. Through two stacked layers of Nafion, each of thickness *t*_N_, the areal resistance is
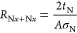
2The Nafion conductivity is dependent on the formulations and preparation of the Nafion. For each type of membrane measured, the Nafion conductivity was determined from eq 1 or 2 from direct measurements of membrane conductance without graphene present.

As has been observed in prior studies, imperfections in graphene lead to transport pathways through the material.^36^ We distinguish between larger tears in the material and smaller defects on the basis of size, which can lead to different implications for transport through the material (Figure 3A). With a graphene layer covering one side of the Nafion, proton transport through the membrane can occur through tears or defects or through the graphene lattice itself. Previous work has found that the conductance of tears and defects is significantly higher than that through the lattice,^36^ so the latter pathway is neglected in this modeling. An equivalent transport resistance model is presented in Figure 3B.

To cross the membrane through a tear, ions experience resistance to funneling toward the tear within the solution (*R*_TAS_), to passing through the tear (*R*_TP_), and to diffusing through the Nafion on the other side. The resistance within the Nafion has two components: (1) resistance to 1D conduction through the material (*R*_TCN_), which is present even without graphene, and (2) spreading resistance (*R*_TSN_) within the Nafion away from the tear, by which ions diffuse out radially from the tear to access a wider area of the Nafion support through which to conduct. The spreading resistance is negligible for membranes that are thick relative to the spacing between tears (Figure 3D, top) but could restrict transport when the membrane is thin compared to this spacing because ions cannot access the entire cross-sectional area to diffuse to the other side (Figure 3D, bottom).

The access resistance and pore resistance are estimated from the analytical equation for ion conduction through a circular opening in a thin plate with the same medium on either side (Supporting Information).

In prior studies of membranes with the same structure and type of graphene, we found that an average defect diameter of 0.8 Å and defect density of 3.3 × 10^10^ cm^–2^ could match the measured conductance and ion selectivity values.^36^ The same values are used in the modeling calculations presented here. Tears can have a range of sizes but are approximated as all being circular with a diameter of 1 μm. We note that different choices of tear diameter and fractional area coverage can be used to obtain similar conductance, but this is a reasonable size expected in graphene and is used to demonstrate that the transport pathways described can account for the measured conductance.

Figure 3E compares model values of areal conductance to measurements, while Figure 3F shows the conductance ratio for the graphene–Nafion composite membranes compared to that of Nafion without graphene. The model bands show predictions for the graphene fractional area occupied by tears between 3.5% and 14%. The significantly greater reduction in conductance with the same graphene on the N10 layers compared to the N211 layers can be ascribed to a lower tear density over N10, potentially due to the embedded reinforcement layer. N5 also has a reinforcement layer, but the overall thinness of the material allows for wrinkling during processing, which may allow the formation of tears, resulting in increased conductance. These observations indicate that subtle changes in membrane fabrication to accommodate differences in the Nafion support have an important effect on the resulting membrane quality. The modeling further demonstrates how transport pathways through tears and defects can account for the experimentally measured conductance.

The pathways of transport for protons and H_2_ through Nafion are different,^4,5,8,12,13,36^ and interfacing CVD graphene can allow for the leveraging of the high proton conductance of selective intrinsic defects as well as reduced H_2_ crossover^4−6,8,36^ due to (i) impermeability of the pristine regions of the 2D lattice and (ii) the low propensity of large defects in 2D materials to precisely overlap with the water channels in the Nafion that allow for H_2_ crossover.^4,13,16,22,56^Figure 2C shows H_2_ crossover measured through each PEM by supplying humidified H_2_ to one side and humidified N_2_ to the other as well as the percent H_2_ crossover reduction (Figure 2D) with respect to the controls. A different trend compared to areal proton conductance (Figure 2A,B) is observed for H_2_ crossover (Figure 2C,D); i.e., ∼20–25 μm thick membranes (N211, N211|G, and N10|G|N10) show the least crossover (0.4–1.1 mA cm^–2^), and 5 μm thick membranes (N5 and N5|G) show the most (∼57.7 mA cm^–2^). However, the greatest percent reduction in H_2_ crossover is observed for the N10|G (∼78%) and N10|G|N10 (∼64%) membranes, which is consistent with the areal conductance measurements, while those of N211|G (∼32%) and N5|G (∼40%) membranes are similar. Interestingly, the H_2_ crossover for the N5|G membrane (∼34.7 mA cm^–2^) is an order of magnitude greater than that of the bare N211 membrane (∼1 mA cm^–2^), and N10|G H_2_ crossover (∼0.7 mA cm^–2^) is similar to N211|G H_2_ crossover (∼0.7 mA cm^–2^), despite N10|G being ∼50% as thick as N211|G, indicating that high-quality graphene is an effective barrier to H_2_. Upon interfacing N10 with FG, the H_2_ crossover increases to ∼2.1 mA cm^–2^, which is consistent with the presence of more defects, including nonselective defects.

## Performance of Nafion|G PEMs in a Custom-Built Cell at Room Temperature

**Figure 4:**
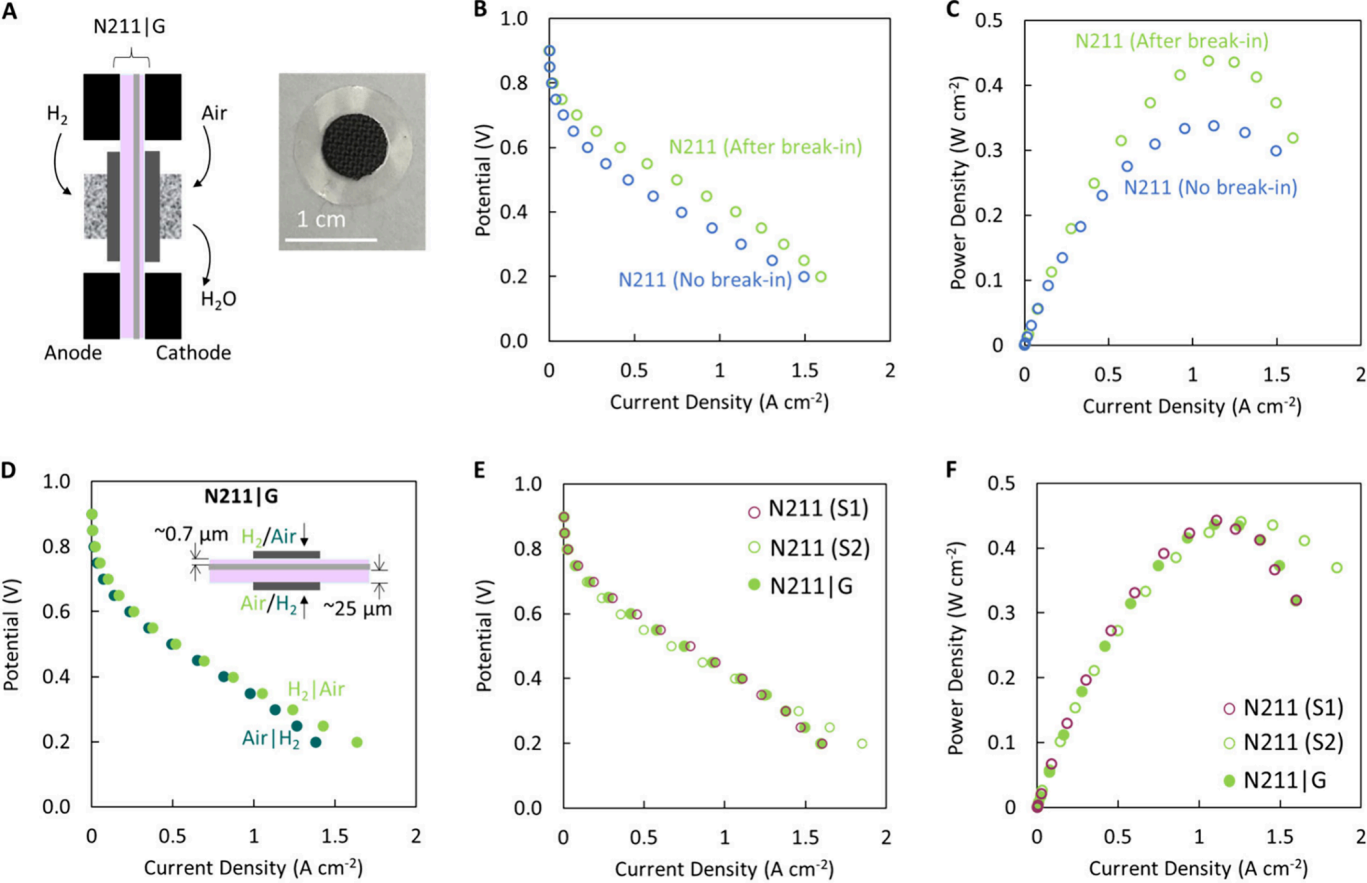
Performance of N211 and N211|G membranes as PEMs in a custom-built cell at room temperature. (A) Schematic of the cell geometry (active area of ∼0.32 cm^2^) and optical image of the MEA with Pt/C-cloth electrodes (loading of 0.2 mg of Pt/cm^2^). The cell is operated at room temperature and atmospheric pressure, with 40 sccm humidified H_2_ supplied to the anode and 120 sccm humidified air supplied to the cathode. (B) Polarization curves and (C) power density curves for the N211 control membrane before and after the break-in process showing an improvement in performance. (D) Polarization curves for the N211|G membrane when the orientation of the graphene is closer to the anode (H_2_, green curve) or the cathode (air, blue curve). See the inset schematic for the orientation of the graphene membrane relative to the input gases. Only marginal differences are observed when the membrane orientation is changed. (E) Polarization curves and (F) power density curves for two separate N211 control membranes (empty red and green circles) and the N211|G membrane (filled green circles). Also, see Figure 2C for H_2_ crossover measurements for these membranes.

Since the N211|G membrane demonstrated the smallest reduction in areal proton conductance (∼7.5%), we evaluate its performance at room temperature using a custom-built cell in comparison to that of N211 (Figure 4). After the break-in procedure, which was used to ensure humidification of the membrane and the stability of the measurements, the current density at 450 mV for N211 increases from 610.9 to 923.9 mA cm^–2^ and the peak power density increases from ∼0.34 to ∼0.44 W cm^–2^ (Figure 4B,C). Next, we evaluated whether the configuration of the N211|G membrane in the cell had an effect, i.e., if the ∼700 nm spin-coated Nafion covering graphene on N211 faced the anode (H_2_ side) or the cathode (air side), and found no significant effect in the polarization curves, regardless of orientation (Figure 4D).

Interestingly, the N211|G membrane showed performance comparable to that of bare N211 measured under similar test conditions [i.e., room temperature and atmospheric pressure (Figure 4E,F)],^61,62^ indicating the potential benefits of interfacing CVD graphene with Nafion for PEMs with high proton conductance and low H_2_ crossover. Electrochemical impedance spectroscopy (EIS) (Figure S3C) is used to measure the high-frequency resistance (HFR). The reduction in HFR for the N211|G membrane from ∼105.3 mΩ cm^2^ (at RT) in the custom-built cell to ∼58.3 mΩ cm^2^ (∼80 °C) in a standard Scribner fuel-cell test station indicates that decreasing the system/contact resistance via controlled compression and increasing the temperature may allow for higher performance (Figure S3A,B), particularly in the region between 0.5 and 0.8 V where ohmic losses dominate. Indeed, subtraction of the ohmic resistance contribution from the polarization curve shows better agreement between the two test systems (Figure S3D).

## Fuel-Cell Performance of Nafion|G PEMs with H_2_/Air and H_2_/O_2_ Fuel Cells at 80 °C and 150–250 kPa-abs

**Figure 5:**
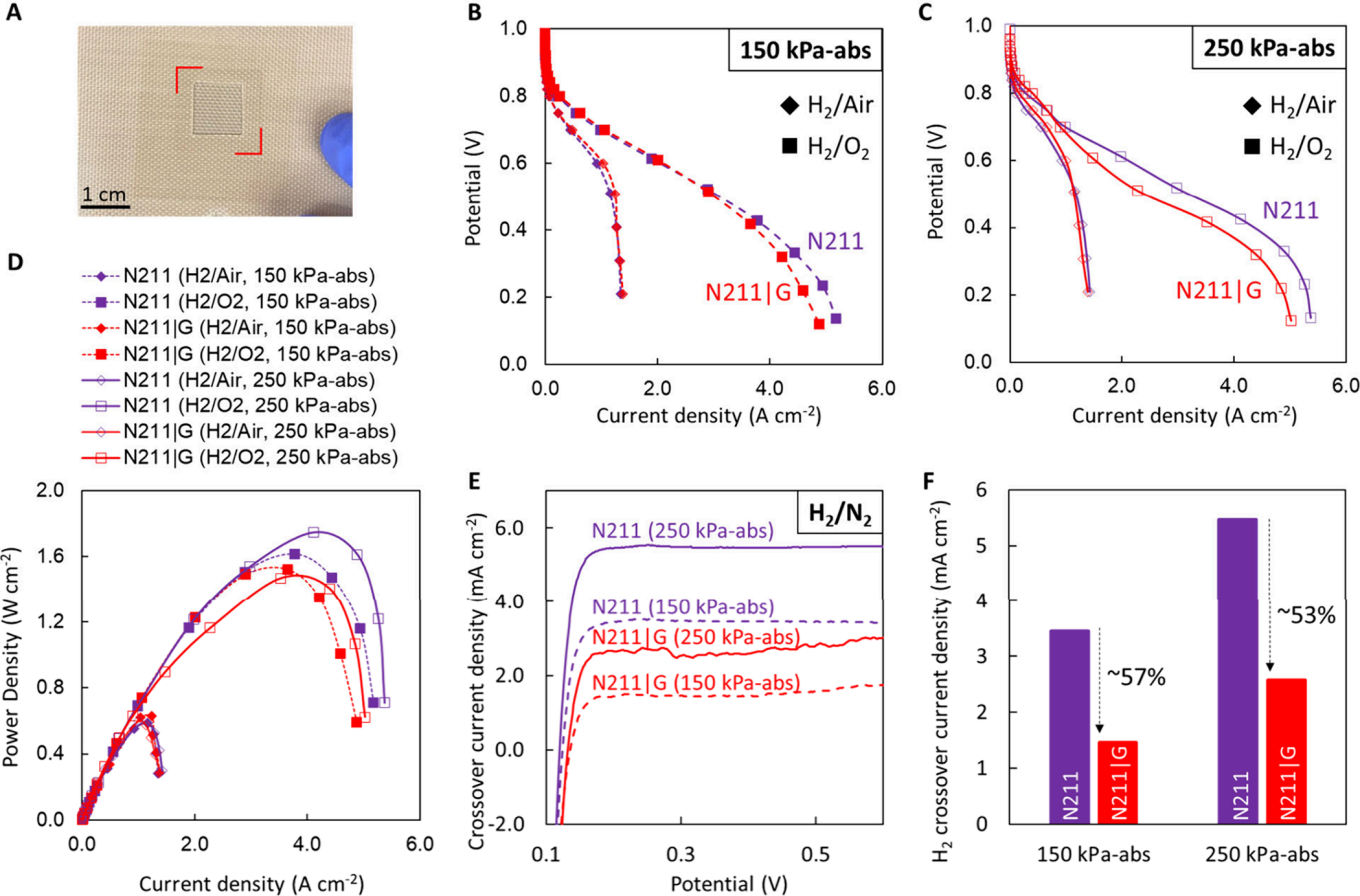
Performance of N211|G membranes operating in an H_2_/air and H_2_/O_2_ fuel cell at 80 °C and different pressures (150 and 250 kPa-abs). (A) Image of the N211|G sample prior to coating with catalyst ink, with graphene corners indicated by red lines. The active area (defined by the Kapton window) for the N211 and N211|G membranes is ∼1 cm^2^. Polarization curves at (B) 150 kPa-abs and (C) 250 kPa-abs with H_2_/air (diamonds) and H_2_/O_2_ (squares) at 80 °C and 100% relative humidity. A similar performance is observed for both N211 and N211|G membranes under most conditions, with the N211|G membrane showing some deviation at a higher pressure with O_2_. (D) Power density curves for N211 (purple, empty symbols) and N211|G (red, filled symbols) membranes with H_2_/air (diamonds) and H_2_/O_2_ (squares) geometries at 150 kPa-abs (filled symbols, dotted lines) and 250 kPa-abs (empty symbols, solid lines). (E) Crossover curves for a N211 control membrane at 150 kPa-abs (purple dotted line) and 250 kPa-abs (purple solid line) and a N211|G membrane at 150 kPa-abs (red dotted line) and 250 kPa-abs (red solid line). (F) Extracted H_2_ crossover current density at 0.4 V from panel E, demonstrating the reduction of H_2_ crossover with the addition of the graphene layer by ∼57% and 53% at 150 and 250 kPa-abs, respectively. Also, see Figure S4 for the electrochemical surface area determined by CO stripping, Figures S5 and S7 for *iR*-corrected Tafel plots, and Figure S6 for *iR*-corrected and roughness factor-normalized polarization curves.

Finally, we evaluate the performance of the N211|G membranes relative to that of bare N211 under conditions relevant to practical fuel-cell operation [80 °C, 100% relative humidity, back pressure ∼150–250 kPa-abs (Figure 5)]. For better compatibility with the system, the transferred graphene area is increased to ∼2 cm^2^, with an active area of ∼1 cm^2^, ensuring that the active area is fully covered by the graphene (Figure 5A).

Beginning-of-life H_2_ crossover is evaluated using LSV after the break-in or the conditioning procedure (see methods and Table S1). With the addition of graphene, the H_2_ crossover current density at 0.4 V is reduced by ∼57% to ∼1.5 mA cm^–2^ compared to that of bare N211 (∼3.4 mA cm^–2^) at a backpressure of 150 kPa-abs (Figure 5E,F). This is an improvement to the ∼34% reduction in H_2_ crossover we observed for a similarly prepared, smaller-area membrane^4,63^ and is attributed to the break-in process that increases crossover for N211. A similar reduction in H_2_ crossover is observed (∼53%) when the back pressure is increased to 250 kPa-abs (Figure 5E,F).

Next, we evaluate the performance of N211|G compared to that of bare N211 using 150 kPa-abs (Figure 5B) of air/H_2_ at the cathode/anode, respectively, and observe the performance of the N211|G membrane to be comparable to that of the bare N211 membrane, reaching maximum current densities of ∼1.3 A cm^–2^ (Figure 5C), which is consistent with the literature.^64−66^

Cell-to-cell variations in the platinum electrochemically active surface area (ECSA), determined by CO stripping (Figure S4), can induce small differences in the kinetic region of the polarization curves. The ECSA-normalized current can eliminate these differences. However, the addition of a graphene layer decreases the H_2_ crossover current, which has the favorable impact of increasing the open circuit voltage (OCV) for N211|G cells, resulting in higher currents in the kinetic region and beyond (Figure S5). The beneficial effect of the graphene layer is notable in the polarization curve with the Pt surface area-normalized current, confirming the higher kinetic performance of N211|G cells (Figure S6). The lower performance of N211|G cells at a higher current density is attributed to the graphene layer impeding the transport of produced water into the membrane, potentially resulting in flooding of the cathode. This results in minor differences in the peak power densities (Figure 5D) between N211 and N211|G, both within the range observed in the literature (∼0.59–0.63 W cm^–2^),^65−67^ suggesting the addition of graphene does not significantly hinder the overall PEM performance when operating in a fuel cell at 80 °C.

Replacement of air with O_2_ at the cathode results in an expected increase in the OCV and Pt surface area-normalized kinetic current (Figure S7). Akin to the performance in air, control N211 membrane and N211|G membrane cells show similar performance in the kinetically dominated region (≥0.7 V) for the O_2_-fed cathode while the ohmic resistance-dominated region shows differences (Figure 5B,C). This difference is reflected in the HFRs for N211|G (∼47.20 mΩ cm2) and N211 (∼45.50 mΩ cm^2^) at 400 mV, and upon iR correction and normalization to the ECSA, the ohmic regions of the polarization curves are in good agreement. Small differences between the N211 and N211|G membranes are observed in the maximum current and power densities (Figure 5B–D). The minor reduction in the maximum current density for N211|G (∼1.52 W cm^–2^) compared to that of the N211 control (∼1.62 W cm^–2^) with back pressure to 150 kPa-abs is exacerbated by an increase in the back pressure to 250 kPa-abs (Figure 5C,D). Compared to the custom-built cell (Figure 4), we observe similar maximum current densities for the N211 and N211|G membranes, but an increase in the maximum power density under the relevant testing conditions (80 °C, 100% relative humidity, 150–250 kPa-abs) (Figure 5). Considering O_2_ transport effects can manifest at >300 mA cm^–2^ as well as reduced H_2_ crossover with graphene (Figure 5E), our experiments potentially suggest the graphene layer could increase transport resistance for water produced at the cathode that would have otherwise diffused into the PEM. Ultimately, for practical applications at lower operation current densities, air feed, and lower back pressures, such effects are expected to be relatively inconsequential.

Taken together, our experiments demonstrate that integrating atomically thin graphene films with Nafion^4,5,8,12,13,36^ can simultaneously allow for high proton conductance by leveraging selective intrinsic defects as well as reduced H_2_ crossover^4−6,8,36^ due to (i) the impermeability of the pristine 2D lattice to atoms and/or molecules and (ii) the low propensity of large defects in CVD-grown 2D materials to precisely overlap with the water channels in the Nafion that typically allow for H_2_ crossover.^4,13,16,22,56^ These observations illustrate the potential for interfacing CVD graphene into centimeter-scale PEMs, run under relevant conditions with an improvement in H_2_ crossover without detriment to performance, thereby offering the opportunity to overcome the typical trade-off between conductance and H_2_ crossover for advancing next-generation PEMs.

In summary, we demonstrate a facile approach to integrate CVD graphene with tunable defect density with Nafion of varying thicknesses (∼5–25 μm) to overcome the persistent gas crossover–conductance trade-off inherent to polymeric fuel-cell PEMs. Notably, incorporating atomically thin monolayer CVD graphene into PEMs allows H_2_ crossover reduction of ∼34–78% compared to that of Nafion of similar thickness while simultaneously maintaining adequate proton conductance for applications (>4 S cm^–2^). Our experimental observations in conjunction with resistance modeling allow for a systematic and improved understanding of the critical trade-offs between the complex interplay of graphene defect density and Nafion proton transport resistance on areal proton conductance of the resulting graphene|Nafion composite membranes as well as H_2_ crossover. Interfacing high-quality low-defect density CVD graphene on Nafion 211 (∼25 μm thickness), i.e., N211|G, allows for the high areal proton conductance (∼6.1 S cm^–2^) as well as the lowest H_2_ crossover (∼0.7 mA cm^–2^). Our fully functional centimeter-scale N211|G fuel-cell PEMs demonstrate performance comparable to that of state-of-the-art Nafion N211 with H_2_/air (power density ∼0.57–0.63 W cm^–2^) and H_2_/O_2_ feed (power density ∼1.4–1.62 W cm^–2^) at room temperature and under standard operating conditions (∼80 °C, ∼150–250 kPa-abs). Future efforts focused on understanding the implication of incorporating CVD graphene into PEMs, including long-term performance and/or efficiency and PEM stability and/or durability, could enable progress toward realistic applications. We expect our work will aid future developments of PEMs for fuel cells, and overcoming the crossover–conductance trade-off is also highly relevant for flow batteries, electrolysis, isotope separation, and other PEM technologies.

## Supporting Information Available

Experimental methods, impact of hot pressing on thin (∼5–10 μm), reinforced Nafion (Figure S1), areal proton conductance for N211|G and N10|G membranes (Figure S2), ohmic resistance losses of N211 and N211|G (Figure S3), CO stripping voltammogram for the cathode catalyst layer (CL) (Figure S4), comparison of *iR*-corrected Tafel plots between graphene-free (N211) and graphene-coated (N211|G) MEAs under H_2_/air conditions at 80 °C and 100% relative humidity (Figure S5), comparison of *iR*-corrected and roughness factor-normalized polarization curves under H_2_/air (Figure S6), comparison of *iR*-corrected Tafel plots between graphene-free (N211) and graphene-coated (N211|G) MEAs under H_2_/O_2_ conditions at 80 °C and 100% relative humidity (Figure S7), summary of the conditioning protocol (Table S1), and calculated *iR*-corrected polarization curves for the N211|G membrane in a custom-built cell (active area of ∼0.32 cm^2^) (Table S2) (PDF)
