# Supplementary material for: Overcoming the Conductance versus Crossover Trade-off in State-of-the-Art Proton Exchange Fuel-Cell Membranes by Incorporating Atomically Thin Chemical Vapor Deposition Graphene
Source: Nano Lett. 2025 Jan 13;25(3):1165–76. doi: 10.1021/acs.nanolett.4c05725 (PMC11760178; doi:10.1021/acs.nanolett.4c05725)
Supplement: Supplementary file 1 — nl4c05725_si_001.pdf [file nl4c05725_si_001.pdf]

Supporting Information to:

Overcoming the Conductance vs Cross-over Trade-off in State-of-the-art Proton Exchange Fuel-Cell Membranes by Incorporating Atomically Thin CVD Graphene

Nicole K. Moehring[1,2,3], Abdul Bashith Mansoor Basha,[4] Pavan Chaturvedi[2,3], Thomas Knight[6], Xiaozong Fan[2], Peter N. Pintauro[2], Michael S. H. Boutilier[5], Kunal Karan[4], Piran R. Kidambi[1,2,3,7,8]*

[1] Interdisciplinary Graduate Program in Materials Science, Vanderbilt University, Nashville, TN 37235.

[2] Chemical and Biomolecular Engineering Department, Vanderbilt University, Nashville, TN 37212.

[3] Vanderbilt Institute of Nanoscale Science and Engineering, Nashville, TN 37212.

[4] Department of Chemical and Petroleum Engineering, University of Calgary, Calgary, AB, T2N 1N4, Canada.

[5] Department of Chemical and Biochemical Engineering, Western University, London, Ontario, N6A 5B9, Canada

[6] Department of Chemistry, Vanderbilt University, Nashville, TN 37235.

[7] Mechanical Engineering Department, Vanderbilt University, Nashville, TN, 37212, United States.

[8] Walker Department of Mechanical Engineering, University of Texas at Austin, TX, United States.

* Corresponding author: piran.kidambi@vanderbilt.edu

The supporting Information includes:
Experimental Methods
Supporting Figures S1-S7
Supporting Tables S1 and S2

Experimental Methods

1. *Graphene growth*

Graphene was synthesized using a custom built hot walled CVD reactor.[1–7] Cu foil (HA, 18 µm thickness, JX Holdings) is cleaned via sonication in 20 v/v% HNO_3 for 4 minutes followed by DI water rinse, air dried, and loaded into the reactor (base pressure ~15 mTorr).[1] The reactor is heated to 1060 °C (~35 °C/min ramp rate) with 100 sccm H_2 (~4 Torr) and the Cu foil is annealed for 60 min.[1]

Two different kinds of graphene are used in this study: 1) high quality graphene (G) with square domains and 2) graphene grown fast with a higher supply of dendritic domains (FG).[1]

Conditions used for G: 1060 °C, 300 sccm H_2 (~14 Torr), growth step #1 with 0.5 sccm CH_4 for 60 min, growth step #2 with 1 sccm CH_4 for 30 min. The reactor is quench cooled to room temperature.[1]

Conditions used for FG: 1060 °C, 100 sccm H_2 (~4 Torr), growth step #1 with 2 sccm CH_4 for 30 min, growth step #2 with 4 sccm CH_4 for 30 min.[1] The reactor is quench cooled to room temperature.[1]

To obtain individual domains of graphene, growth times of ~5 min for G (Figure 2E) and 30 s for FG are used (Figure 2G).[1]

2. *Electrochemical etch test*

Electrochemical etch test is used to estimate the defect density of the synthesized G and FG.[1,4,5,8] In a two-electrode geometry, the working electrode connected to graphene on Cu foil (~0.5 × 1 cm^2) and the reference/counter electrode connected to bare Cu foil (~1 × 5 cm^2) were submerged in 0.5 M $CuSO_4$ and a 1 V bias was applied between the electrodes for 1 s. The graphene on Cu foil was then immediately rinsed in DI water, dried, and imaged with SEM (Figure 2F, H). Etched regions underneath defects in graphene appear as bright, white spots in SEM images and ImageJ software was used to calculate the total area etched.

3. *Raman spectroscopy*

Raman spectra (Figure 1E) were obtained using a ThermoFisher DXR Confocal Raman Microscope (532 nm laser, 1-3 mW power). Raman spectrum of graphene on spin coated Nafion is obtained by using a Si/SiO_2 wafer (300 nm SiO_2) as a support, by gently pressing the wafer downward on the floating G|Nafion such that a Si/SiO_2/Nafion/Graphene stack is obtained with graphene on the top side.

The spin coated Nafion control is obtained following the spin coating process described above but on bare, annealed Cu foil and the free-standing Nafion scooped onto Si/SiO_2 wafer.

For Raman of CVD graphene on Si/SiO_2, graphene is transferred via poly-methyl methacrylate (PMMA) transfer.[1,4,6,8,9] Briefly, PMMA solution (MW 35000, 2 wt % in anisole) is drop casted on graphene and dried at room temperature before etching the Cu foil in 0.2M ammonium persulfate (APS) as described above. The G|PMMA stack is scooped onto Si/SiO_2 wafer and dried (30 min at 60 °C). The PMMA is removed by soaking in acetone (~12 h) and rinsing in isopropanol.

4. *Graphene transfers via spin coated Nafion and membrane fabrication*

Nafion is spin-coated on CVD graphene on Cu foil (~1.5 × 1.5 cm^2) in 3 steps:

Step 1: ~0.5 mL of Nafion dispersion (5 wt%, Alcohol based, 1100 EW, Ion Power), 1000 RPM, 60 s solvent evaporation at 60 °C for 10 min.

Step 2: ~0.5 mL of 2.5 wt% Nafion dispersion (diluted from 5 wt% solution with ethanol), 1000 RPM, 60 s, solvent evaporation at 60 °C for 10 min.

Step 3: ~0.5 mL of 2.5 wt% Nafion dispersion, 1000 RPM, 60 s, final bake at 60 °C for 30 min resulting in ~700 nm spin-coated Nafion film (cross-sectional SEM image in Figure 1C).

The Nafion coated graphene on Cu is pre-etched by floating on 0.2 M APS solution for 10 min, followed by a DI water bath and removal of any graphene on the back side via laboratory wipes. The Cu foil is completely etched in 0.2 M APS solution (~4 h) and the G|spin-coated-Nafion stack is rinsed by floating it on DI water.

N10 and N211 substrates (~2 × 2 cm^2) are prepared by adhering to PTFE-coated fiberglass supports (9 mil thickness) via mild hot press (~130 °C, ~200 PSI, ~30 s) then used to scoop out the G|Spin-coated-Nafion stack from a DI water bath.

N5 membranes are not hot pressed (see Figure S1) but rather suspended over a PTFE-coated fiberglass donut (~1/2” OD, ~3/8” ID) and then used to scoop out the G|spin-coated-Nafion stack, as the N5 membranes are highly sensitive to hot pressing.

The membranes are dried at ~60 °C for ~12 h before adding Pt/C electrodes (~0.2 mg Pt/cm^2 on carbon cloth, ~0.32 cm^2, Fuel Cell Store) to both sides via hot press (~140 °C, ~200 psi, ~1 min).

For the N10|G|N10 sandwich membrane, an additional N10 layer is hot pressed to the N10|G stack (~140 °C, ~400 psi, ~1 min) before the electrodes are added. Due to the fragility of the N5 membranes, PTFE-coated-fiberglass gaskets are used to provide support and minimize the formation of tears (Figure S1B, C).

5. *Areal Proton Conductance, H_2 crossover and fuel cell at room temperature ~20-25°C*

Proton transport through the fabricated membranes is measured at ambient conditions (room temperature and atmospheric pressure) by supplying humidified H_2 gas ~40 sccm to both sides of the membrane (symmetric H_2 gas feed, humidified through H_2O bubblers) loaded into a custom-built test cell using rubber O-rings to seal and isolate the gas flow on either side of the membrane. Porous Ni foam is used to make electrical contact between the electrodes and graphite current collectors. A Gamry 1010E potentiostat collects linear sweep voltammetry (LSV), sweeping from -100 mV to +100 mV (scan rate 2 mV s^{-1}) while measuring current. Current density is calculated by dividing the measured current by the electrode area (~0.32 cm^2). I-V curves are plotted as potential versus current density (Figure 2A) and the areal proton conductance is extracted by taking the inverse of the slope using Ohm’s law (Figure 2B).

H_2 crossover is also measured using the same custom-built test cell (per DOE protocol).[1,10,11] 40 sccm H_2 flows to one electrode (reference/counter electrode) and 40 sccm N_2 to the other (working electrode). Once the open circuit potential is stable (~90-120 mV), LSV is run from 700 mV to -150 mV (Figure 2C). H_2 crossover current density is taken at 400 mV (Figure 2D), as this region is free from effects of the Pt catalyst (*i.e.* H^+ adsorption/desorption) and used to compute % reduction relative to the respective controls.

Polarization curves for fuel cell operation in the custom-built test cell were obtained at room temperature while flowing 40 sccm H_2 to one electrode and 120 sccm air to the other (Figure 4). A 1:3 ratio of H_2:air is used to compensate for the higher oxygen consumption at a specific current compared to hydrogen (per Faraday’s law) and mass fraction of O_2 in air (~23%).[12] The potential is swept from 900 mV to 200 mV stepwise (50 mV step size, 60 s per step).[13,14] A break-in step by cycling between 200 mV and 600 mV (60 s hold each, repeat ~100×) until the current stabilizes is used to condition the membrane.[14,15] The additional

~700nm thick Nafion layer's influence is evaluated by swapping the H_2/air inlets (Figure 4D). Power density is calculated by multiplying the current density by the set potential.

Electrochemical impedance spectroscopy (EIS) is used to determine the membrane/contact resistance (HFR, Figure S3, 20 kHz to 1 Hz, AC voltage 10 mV rms, DC voltage 450 mV, 20 points per decade) from the intersection of the Nyquist plot with the x-axis. The intersection point is estimated by fitting the EIS in the Gamry Analyst software with a fuel cell equivalent circuit. To compare polarization curves without the influence of the high ohmic resistance of the custom-built cell, iR correction is performed by multiplying the HFR obtained from EIS with the measured current at each potential, then adding it to the set potential to obtain an iR corrected voltage which is plotted against current density to obtain an iR corrected polarization curve (Figure S3 and Table S2).

6. *Conductance modeling*

The access resistance to reach the pore from the solution side is calculated as,[16]

$$R_{TAS} = \frac{1}{2D_T\sigma_S} \tag{3}$$

and is half the access resistance for a thin pore with the same medium on either side. Here σ_S (S m^{-1}) is the ion conductivity of the solution, and D_T (m) is the tear diameter. The resistance for passing through the tear in the graphene is,

$$R_{TP} = \frac{4t_G}{\pi\sigma_N D_T^2} \tag{4}$$

where $t_G = 6.8$ Å is the graphene thickness.[17] Although the membrane may have a distribution of tear sizes, in this modeling we approximate tears as all having the same effective diameter with the aim of confirming that reasonable values of parameters such as tear size and density can explain the experimental results. The one-dimensional diffusion resistance in the Nafion after a tear is,

$$R_{TCN} = \frac{t_N}{\sigma_N L_T^2} \tag{5}$$

where L_T [m] is the average spacing between the centers of tears. The tear density can be equivalently expressed either in terms of the average spacing between tears (L_T) or the open area fraction occupied by tears (a) of diameter D_T, where the two are related by,

$$L_T = \sqrt{\frac{\pi}{4a}}D_T \tag{6}$$

The spreading resistance within the Nafion away from a tear is approximated from an analytical expression for the spreading resistance from a circular opening at uniform concentration into a circular tube with zero flux on all edges except the opening and the opposite circular face.[18] These boundary conditions approximate an impermeable graphene layer surrounding the tear and a symmetry condition at the midplane between pores. Although the circular boundary does not match the exact geometry, it provides a reasonable estimate given that the membrane structure parameters used in these calculations are also approximate. The spreading resistance is computed as,

$$R_{TSN} = \frac{4L_T}{\pi\sigma_N D_T^2}\sum_{i=1}^{\infty}\frac{1}{\delta_i^3 J_0^2(\delta_i)}J_1\left(\delta_i\frac{D_T}{L_T}\right)\sin\left(\delta_i\frac{D_T}{L_T}\right)\tanh\left(\delta_i\frac{2t_N}{L_T}\right) \tag{7}$$

where J_0 and J_1 are Bessel functions of the first kind of zeroth and first order, respectively, and δ_i are the roots of J_1.

These transport resistances act in series through tears and comprise the left branch in Figure 3B. The combined resistance to passing from solution on one side, through a tear, to solution on the other side is,

$$R_T = R_{TAS} + R_{TP} + R_{TSN} + R_{TCN} \quad (8)$$

The right branch of Figure 3B accounts for transport through smaller defects. Every defect will have diameter dependent resistances to accessing the defect from solution (R_{DAS}), getting through the defect (R_{DP}), spreading out from the pore in Nafion (R_{DAN}), then conducting through the Nafion (R_{DCN}). The access resistance to reach a defect of diameter D [m] from solution is estimated as,

$$R_{DAS} = \frac{1}{2D\sigma_S} \quad (9)$$

The resistance to crossing through the defect in graphene is,

$$R_{DP} = \frac{4t_G}{\pi\sigma_N D^2} \quad (10)$$

The access resistance to spread from a defect in Nafion is approximated as,

$$R_{DAN} = \frac{1}{2D\sigma_N} \quad (11)$$

Conduction in the Nafion from a single defect is approximated as occurring over the average membrane area in which a single defect is found. The average spacing between defects, L_D [m], is calculated from the defect density, n [m^{-2}], as

$$L_D = \sqrt{1/n} \quad (12)$$

The resistance to conduction in Nafion through a single defect is then,

$$R_{DCN} = \frac{t_N}{\sigma_N L_D^2} \quad (13)$$

These resistances act in series through a single defect, resulting in a combined resistance through the defect of,

$$R_D(D) = R_{DAS} + R_{DP} + R_{DAN} + R_{DCN} \quad (14)$$

which depends on the defect diameter. Defects have a range of sizes, approximated by an exponential distribution,

$$p(D) = \frac{1}{\bar{D}} e^{-D/\bar{D}} \quad (15)$$

where p is the probability density for a pore having diameter D, and $\bar{D}$ is a parameter determining the mean and spread of the distribution.[19] Defects act in parallel, resulting in an average conductance (inverse resistance) through defects of,

$$\langle R_D^{-1} \rangle = \int_0^\infty \frac{p(D)}{R_D(D)} dD \quad (16)$$

The net ion conduction through the membrane occurs through tears and defects, which act in parallel. Adding the multiple parallel tears and defects results in an areal conductance of,

$$\frac{G}{A} = \frac{1}{RA} = \frac{R_T^{-1}}{L_T^2} + n(1-a)\langle R_D^{-1} \rangle \quad (17)$$

where G [S] is the total membrane conductance and R [Ω] is the total membrane resistance.

The model is extended to membranes that have Nafion on both sides of the graphene by changing the access resistances in solution to the access and one-dimensional conduction resistances in Nafion (Figure 3C),

$$R_T = R_{TP} + 2R_{TSN} + 2R_{TCN} \tag{18}$$

and

$$R_D(D) = R_{DP} + 2R_{DAN} + 2R_{DCN} \tag{19}$$

Eqn. 18 and 19 replace 8 and 14 in 16 and 17 when computing the areal conductance of membranes with Nafion on both sides of the graphene.

7. Membrane performance in fuel cell at 80°C and different pressures (150 and 250 kPa-abs)

Membrane Electrode Assembly (MEA) Preparation:

The transferred graphene layer is increased to ~2 cm^2, with active area ~1 cm^2. The layered graphene membranes are then loaded into the cell and run at 100% RH, 80 °C, and at 150 kPa-abs and 250 kPa-abs.

The decal transfer method was used to prepare MEAs with active area ~1 cm^2. Catalyst inks for both the anode and cathode were prepared by dispersing Pt/Vulcan catalyst (TEC10V40E, Tanaka Kikinzoku Kogyo K.K., TKK) in a low equivalent weight perfluorosulfonic acid (PFSA) ionomer (3 M, 725 EW). The ionomer-to-carbon (I/C) ratio was maintained at 0.8, while the solid-to-liquid (S/L) ratio was adjusted to 0.1 by adding a mixture of isopropanol (IPA) and ultra-pure water to ensure proper dispersion and optimal ink formulation. The ink components were placed in a 15 mL capped HDPE bottle containing ZrO_2 beads (Glenn Mills, USA) as a grinding medium. The mixture was then processed on a roller mill at 70 RPM for 18 h at room temperature to achieve thorough homogenization.

To create the catalyst-coated decal (CCD), the homogenized catalyst ink was coated onto a virgin PTFE sheet using an automatic film coater (MSK AFA-II, MTI Corporation, USA) fitted with a doctor blade. The coating process was carried out at a controlled speed of 10 mm s^{-1} to ensure uniform deposition. The platinum loading on the cathode and anode electrodes was approximately 0.25 mg_{Pt} cm^{-2}_{geo}, calculated based on the Pt content of the catalyst, I/C of 0.8 and weighing the decals before and after the hot-press process. The catalyst-coated membrane (CCM) with a geometrical area of 1 cm^2 on both the anode and cathode was controlled by a Kapton window created by hot-pressing the CCD against the Nafion-211 and Nafion-211|G|spin-coated Nafion membrane at 150 °C for 3 min with an applied force of 0.12 kN cm^{-2}.

Fuel cell testing:

Fuel cell testing utilized a Biologic SP-200 potentiostat and a 100 W G20 Greenlight Innovation test station (Greenlight Innovation Corp., Canada). Electrochemical measurements were conducted using single-cell equipment sourced from Fuel Cell Technologies Inc. (USA), featuring a 50 cm^2 geometric graphite flow field with a 16 cm^2 channel area.

To achieve approximately 25% compression of the Gas Diffusion Layer (GDL) with microporous layer (MPL) (1.5 cm × 1.5 cm on both sides), the hot-pressed Catalyst-Coated Membrane (CCM) was positioned

between two GDLs containing a microporous layer (250 μm, Toray, TGP-H-060; Fuel Cell Store, USA) and guided by a 175 μm PTFE gasket. To prevent degradation at the edges and to independently control the compression of the catalyst layer and the gas diffusion layer containing the microporous layer (GDL/MPL), the GDL/MPL area was intentionally designed to be larger than the active area of the CCM. During cell assembly, a torque of 30 in-lb was applied in three steps (10 in-lb, 20 in-lb, and 30 in-lb).

Conditioning protocol:

Before testing, all cells underwent conditioning to activate the MEA, hydrate the ionic network, and eliminate potential contamination. The conditioning protocol employed in this study combines elements from the USFCC[20], DOE[21], and NREL(USA)[22]. Specifically, it involves H_2 pumping[23], a constant voltage hold at 0.6 V from UFSCC[20], and a potential cycling conditioning protocol (OCV to 0.6 V) similar to DOE[21] (Table S1). The complete conditioning protocol is outlined in the table below, while a detailed description of each step can be found in our prior publication[24].

ECSA determination by CO-Stripping voltammetry:

CO-Stripping voltammetry was conducted following a protocol similar to that described by Takeshita et al.[25] at 80 °C, 100% RH, and ambient pressure. Prior to testing, the cathode was cleaned by potential cycling between 0.115 V vs. RHE and 0.94 V vs. RHE at a scan rate of 50 mV s^{-1}. CO-Stripping was performed by supplying 0.2 NLPM of a humidified mixture containing 5% CO with N_2 on the cathode and 0.05 NLPM of 10% H_2 with N_2 on the anode. The low percentage of hydrogen on the anode side is to minimize H_2 crossover, which could introduce significant artifacts to the data.[26] During flow conditioning, the cathode was held at 0.08 V vs. RHE for 20 minutes, followed by a 45 min purge with N_2 on both the cathode and anode to remove any residual CO. Subsequently, the cathode potential was scanned from OCV to 0.94 V vs. RHE at a scan rate of 20 mV s^{-1} and held at the final potential to oxidatively strip all electrochemically available CO adsorbed on the Pt surface under the flow of N_2. The scanning process was repeated three times to serve as a baseline and to verify complete oxidation of the adsorbed CO (Figure S4). The integration between the first anodic scan and the third scan was used to determine the Pt ECSA using a specific charge of 420 μC cm^{-2}_{Pt}.

H_2 crossover:

Electrochemical H_2 crossover was assessed via linear sweep voltammetry (LSV) with the anode and cathode pressures maintained at 150/250 kPa-abs under H_2/N_2 (0.1/0.2 NLPM) conditions at 80 °C, 100% RH. The LSV scan ranged from 0.05 V vs. RHE to 0.6 V vs. RHE at a scan rate of 5 mV s^{-1}. To avoid shorting between the anode and cathode, the current was monitored until it reached a steady state between 0.4 V vs. RHE and 0.6 V vs. RHE. The current measured at 0.4 V vs. RHE was then utilized to determine the hydrogen crossover current.

Polarization curves:

Differential flow polarization curves were generated using potentiostatic mode scanning from Open Circuit Voltage (OCV) to 0.8 V to determine kinetic parameters. The kinetic current was recorded over a 2 min period by maintaining a specific voltage within the aforementioned range, with the voltage decreasing in approximately ~20 mV decrements. Subsequently, the remaining current was recorded from 0.8 V vs. RHE to 0.1 V vs. RHE (until limiting current), with a step size of 0.1 V. All experiments were conducted at 80 °C, 100% RH, with a backpressure of either 150 or 250 kPa-abs. A constant flow of 0.3 NLPM of H_2 was maintained on the anode, while 0.5 NLPM of either pure O_2 or air was supplied to the cathode, with no

pressure drop between the anode and cathode. Each polarization point was allowed to stabilize at a specific applied voltage for 3 min, and the resulting equilibrated current values were averaged over the final 30 s.

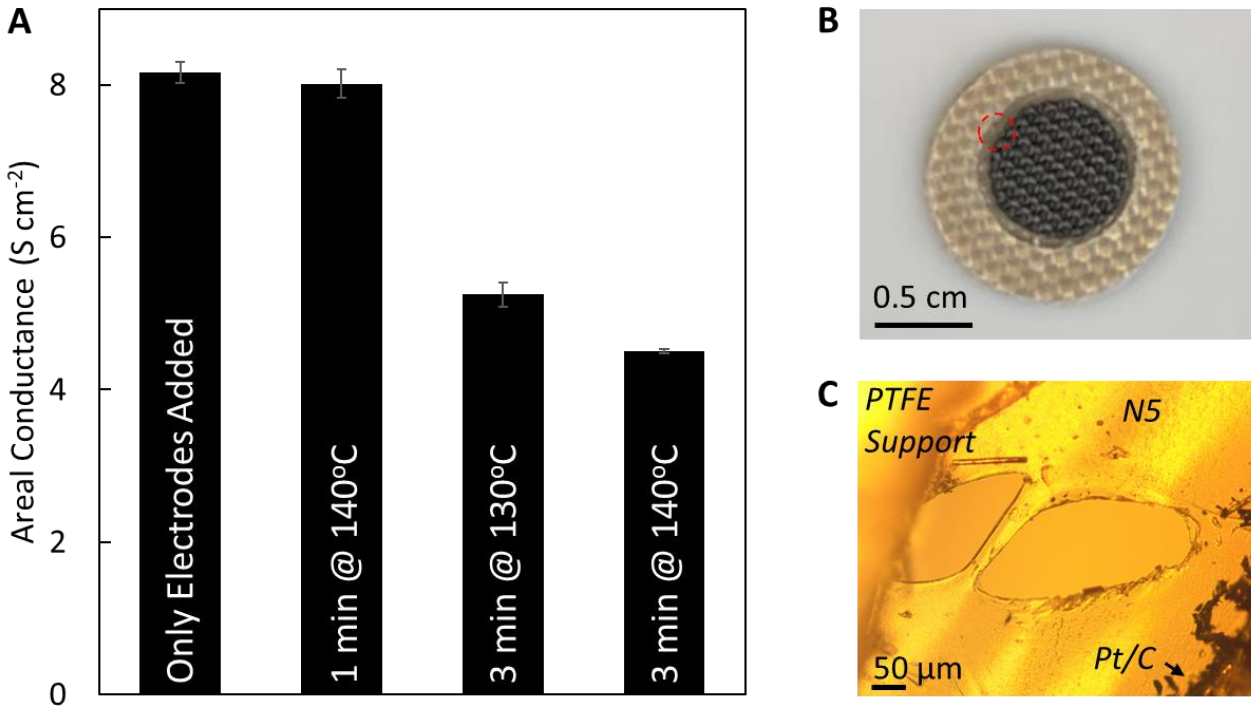

Figure S1. Impact of hot pressing on thin (~5-10 μm), reinforced Nafion.
A) Areal conductance of ~10 μm thick PTFE reinforced Nafion film (N10) after hot press at different conditions (200 psi, 130-140 °C, 1-3 min). Pt/C electrodes are added at 200 psi, 140 °C, for 1-3 min (only electrodes added). Hot pressing N10 for 1 min at 140 °C at 200 psi does not impact areal conductance adversely. However, when the pressing time is increased to 3 min, at ~140 °C or ~130 °C, the areal conductance drops significantly. B) Optical image of a N5 membrane (transparent film), with PTFE-coated fiberglass supports underneath (brown circle in image) and Pt/C electrodes (black circle in image) on top. The region between the PTFE support and the Pt/C is prone to tearing (dotted red circle). C) These tears from hot pressing are visible via optical microscope images.

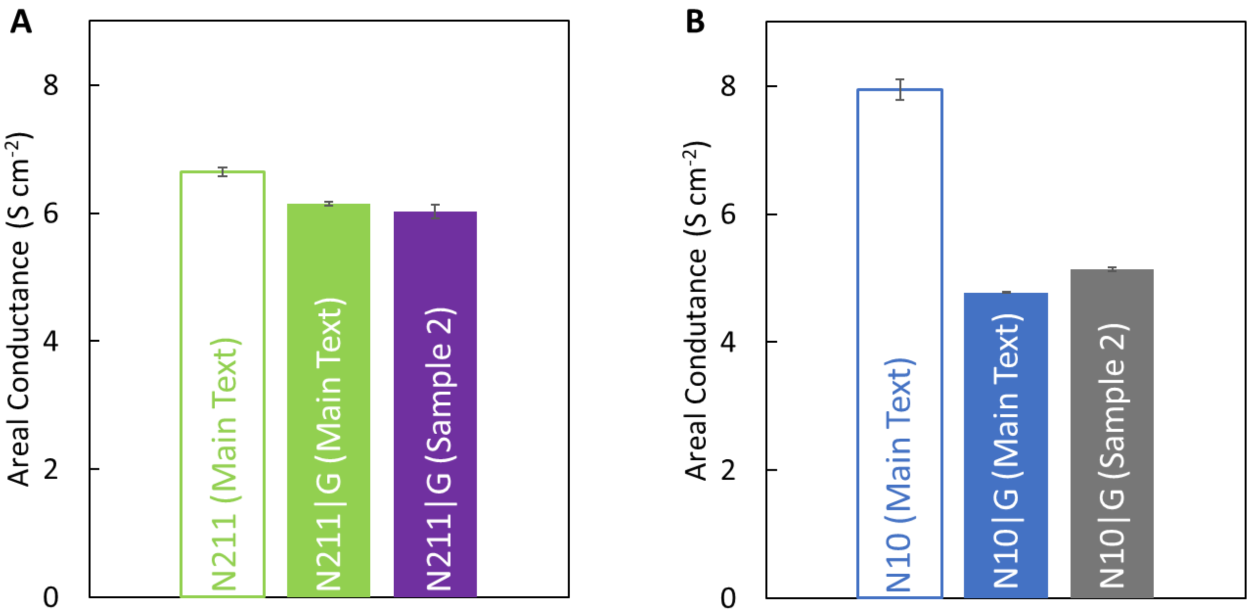

Figure S2. Areal proton conductance for N211|G and N10|G membranes.

A) Areal conductance for two separate N211|G membranes compared to the control membrane (without graphene). Both membranes show a reduction of ~0.5-0.6 S cm^{-2} in areal proton conductance from the addition of CVD graphene. B) Areal conductance measurements for two separate N10|G membranes compared to a single layer N10 control membrane (without graphene). For all N10|G membranes, the drop in conductance from the addition of graphene (~2.8-3.1 S cm^{-2}) is significantly higher than observed for N211|G membranes.

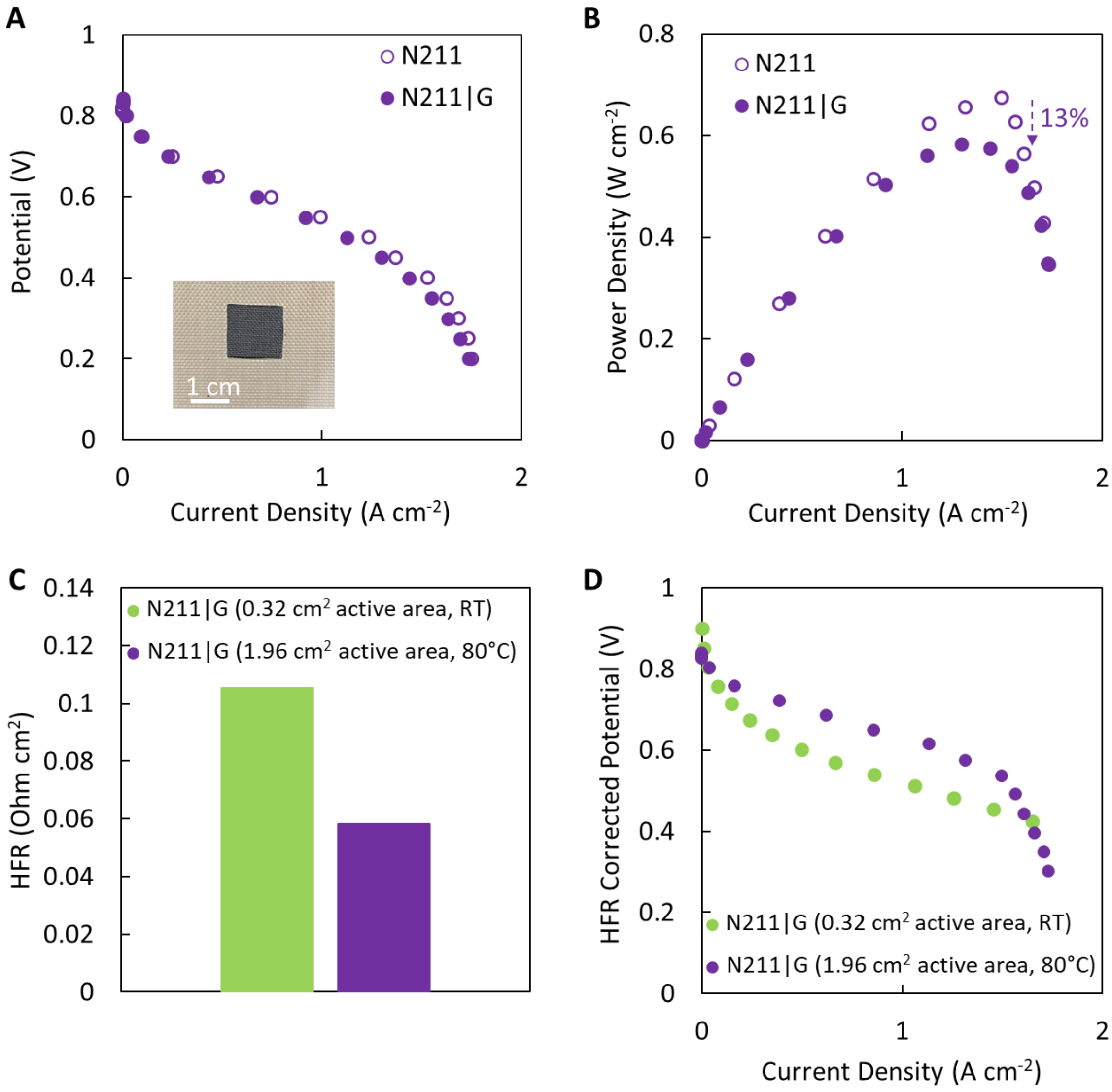

Figure S3. Ohmic resistance losses for N211 and N211|G.

A) Polarization curves and B) power density plots for N211 (open circles) and N211|G (closed circles) membranes after breaking-in (voltage cycling between 0.6 and 0.2 V until current stabilizes, ~100 cycles) measured using a Scribner test station (850E Test System, 80 °C, 160 kPa-abs, 125 sccm H_2, 500 sccm air, active area ~1.96 cm^2). ~2×2 cm^2 graphene area is transferred to N211 using the methods previously described and PTFE gaskets are used to limit the active area to ~1.4×1.4 cm^2 (Figure S3A inset). Polarization curves are collected by voltage sweep from 0.2 V to OCV at intervals of 0.05 V and holding for 1 min at each step. The addition of graphene results in ~13% drop in peak power density compared to the control. C) Comparison of high frequency resistance (HFR) from electrochemical impedance spectroscopy (EIS) at 450 mV for the custom-built test cell (room temperature, RT) and the Scribner test station (80 °C) indicates the custom-built cell has higher ohmic resistance contributions. Note for the Scribner station, the HFR is measured at each voltage step of the polarization curve but the value at 450 mV is used for comparison and HFR correction. D) HFR corrected polarization curves for N211|G membranes measured in the custom-built cell and Scribner test station (see Table S2). While the custom-built cell allows for quick characterization of successful graphene transfer to Nafion, limitations in performance arise due to the increased ohmic losses and ambient temperature operation.

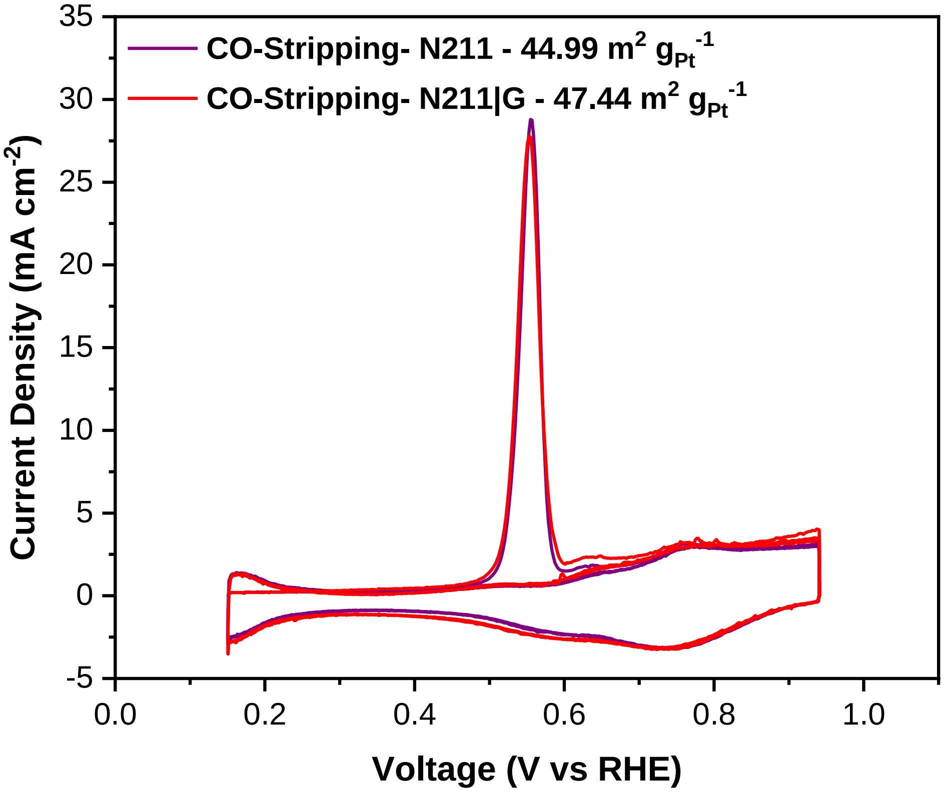

Figure S4. Typical CO stripping voltammogram for the cathode catalyst layer (CL) of graphene-free (N211) and graphene-coated (N211|G) MEAs at 80 °C, 100% relative humidity (RH), and ambient pressure, recorded at a scan rate of 20 mV s^{-1}. The active area defined by the Kapton window for both N211 and N211|G membranes is ~1 cm^2.

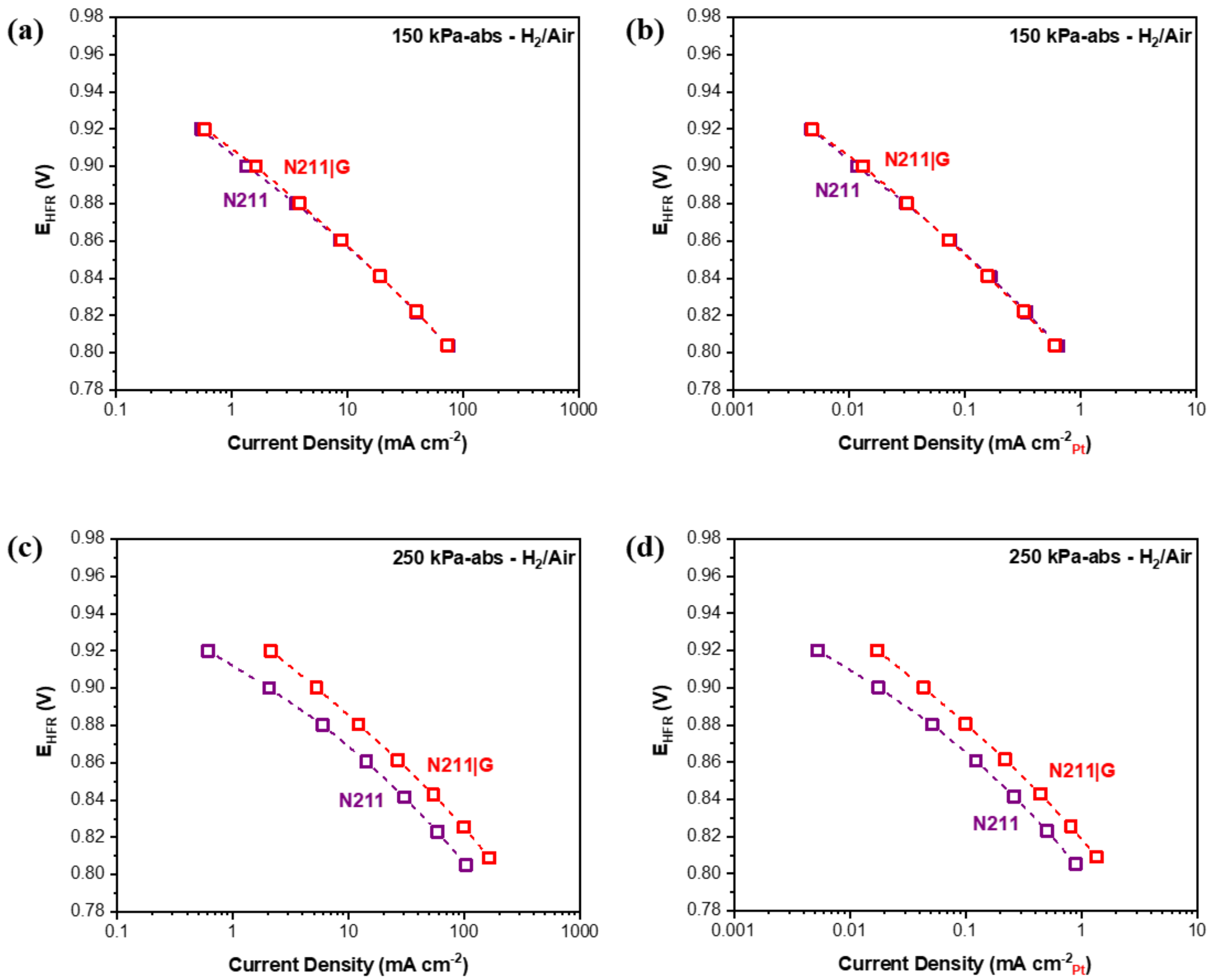

Figure S5. iR-corrected Tafel plot comparison between graphene-free (N211) and graphene-coated (N211|G) MEAs under H_2/Air conditions at 80 °C and 100% relative humidity (RH) at two different pressures: (a) 150 kPa-abs, electrode area-normalized current density; (b) 150 kPa-abs, Pt surface area-normalized current density; (c) 250 kPa-abs, electrode area-normalized current density; (d) 250 kPa-abs, Pt surface area-normalized current density. The active area defined by the Kapton window for both N211 and N211|G membranes is ~1 cm^2.

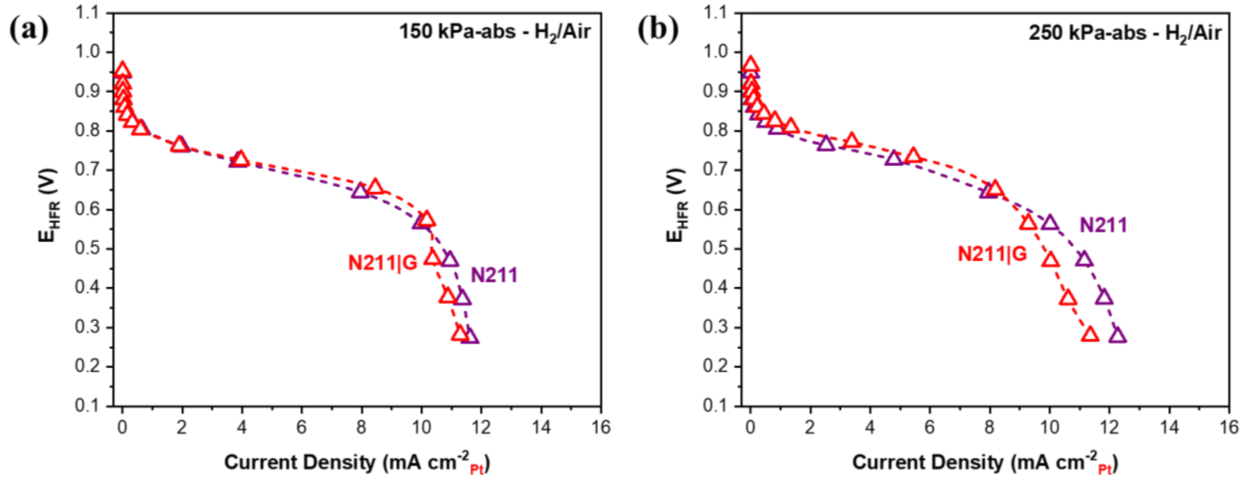

Figure S6. iR-corrected and roughness factor-normalized polarization curve comparison under H_2/air conditions between graphene-free (N211) and graphene-coated (N211|G) MEAs at 80 °C and 100% relative humidity (RH), under (a) 150 kPa-abs and (b) 250 kPa-abs backpressure conditions. The active area defined by the Kapton window for both N211 and N211|G membranes is ~1 cm^2.

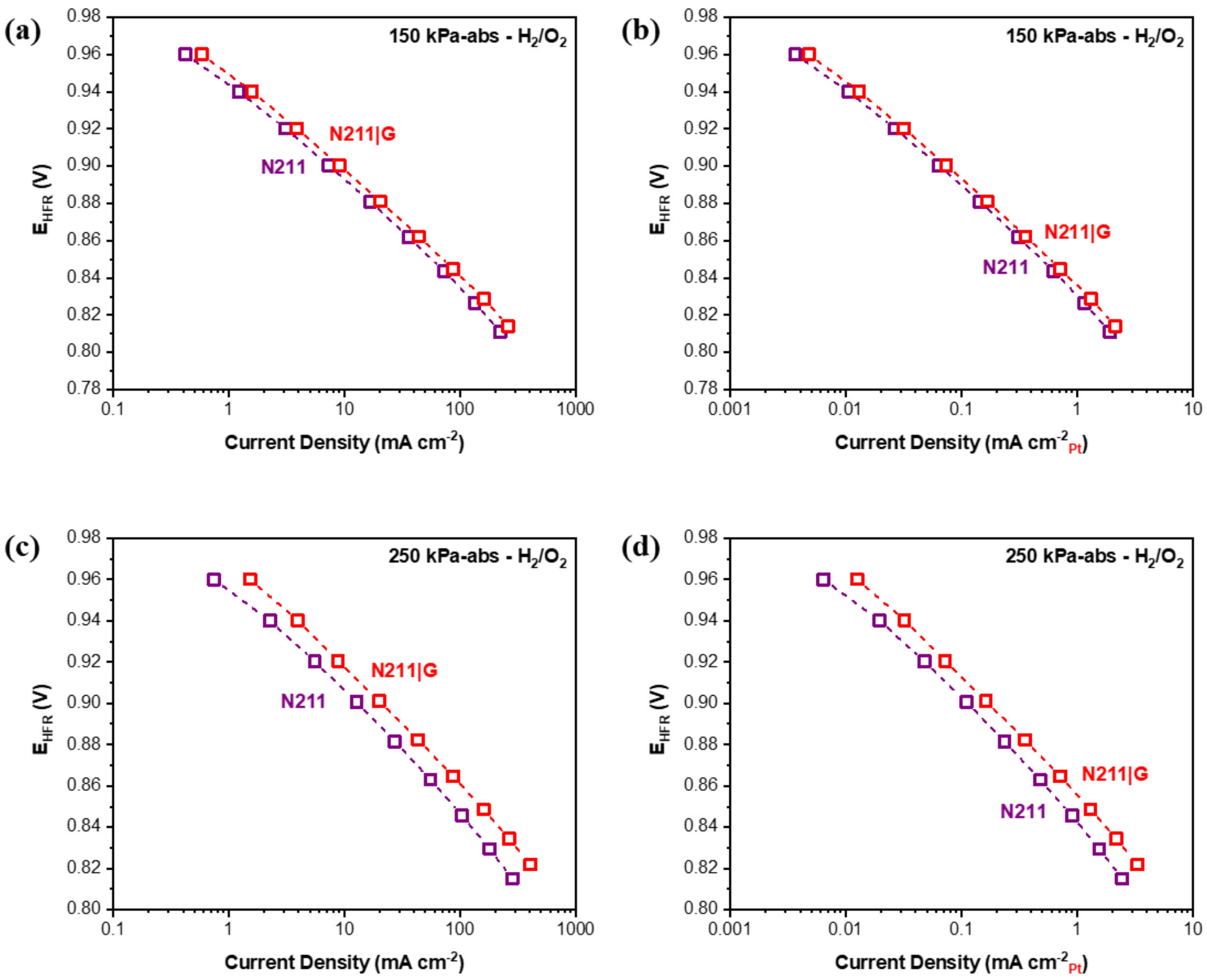

Figure S7. iR-corrected Tafel plot comparison between graphene-free (N211) and graphene-coated (N211|G) MEAs under H_2/O_2 conditions at 80 °C and 100% relative humidity (RH) at two different pressures: (a) 150 kPa-abs, electrode area-normalized current density; (b) 150 kPa-abs, Pt surface area-normalized current density; (c) 250 kPa-abs, electrode area-normalized current density; (d) 250 kPa-abs, Pt surface area-normalized current density. The active area defined by the Kapton window for both N211 and N211|G membranes is ~1 cm^2.

Table S1: Summary of the conditioning protocol.

Step	Name	Inlet Temperature (^{O}C)	Dew point temperature (^{O}C)	RH (%)	Flowrate An/Ca (NLPM)	Reactant gas An/Ca	Absolute Pressure (kPa)	Duration (hrs)
1	H_2 pumping (Current applied: 200 mA cm^{-2})	30	45	~226	0.5/0.5	H_2/H_2	90	0.5
2	Flooding	70	80	~150	0.1/0.2	H_2/N_2	150	8
3	0.6 V hold	80	80	100	0.3/0.5	H_2/O_2	300	~12
4	Potential cycling (OCV- 0.6 V)	80	80	100	0.3/0.5	H_2/O_2	150	Until the last two measured current values are less than 10mA/cm^2

Table S2. Calculated iR corrected polarization curves for N211|G membrane in custom-built cell (active area ~0.32 cm²).

N211|G (~0.32 cm^2 active area)				
Resistance from EIS (HFR, ohms)	3.31×10^{-1}			
Potential (mV)	Current (mA)	Current Density (mA cm^{-2})	Voltage loss (iR drop) between WE and RefE (mV)	iR corrected Voltage (mV)
9.00×10^{2}	5.00×10^{-1}	1.56	1.65×10^{-1}	9.00×10^{2}
8.50×10^{2}	3.11	9.72	1.03	8.51×10^{2}
8.00×10^{2}	1.04×10	3.25×10^{1}	3.44	8.03×10^{2}
7.50×10^{2}	2.48×10	7.75×10^{1}	8.21	7.58×10^{2}
7.00×10^{2}	4.64×10	1.45×10^{2}	1.54×10^{1}	7.15×10^{2}
6.50×10^{2}	7.56×10	2.36×10^{2}	2.50×10^{1}	6.75×10^{2}
6.00×10^{2}	1.13×10^{2}	3.52×10^{2}	3.72×10^{1}	6.37×10^{2}
5.50×10^{2}	1.59×10^{2}	4.97×10^{2}	5.26×10^{1}	6.03×10^{2}
5.00×10^{2}	2.14×10^{2}	6.67×10^{2}	7.06×10^{1}	5.71×10^{2}
4.50×10^{2}	2.75×10^{2}	8.58×10^{2}	9.08×10^{1}	5.41×10^{2}
4.00×10^{2}	3.39×10^{2}	1.06×10^{3}	1.12×10^{2}	5.12×10^{2}
3.50×10^{2}	4.03×10^{2}	1.26×10^{3}	1.33×10^{2}	4.83×10^{2}
3.00×10^{2}	4.65×10^{2}	1.45×10^{3}	1.54×10^{2}	4.54×10^{2}
2.50×10^{2}	5.28×10^{2}	1.65×10^{3}	1.74×10^{2}	4.24×10^{2}
2.00×10^{2}	5.92×10^{2}	1.85×10^{3}	1.96×10^{2}	3.96×10^{2}
			(Voltage loss = iR)	(Potential + Voltage loss)

The resistance contribution due to the membrane and contact (green) is extracted from the EIS at 450 mV (Figure S3C) and used to correct for iR drop. Polarization curves are obtained by sweeping from ~900 mV (OCV) to 200 mV (column 1) and measuring the potential at each step (column 2). The voltage loss due to ohmic resistance (i.e. the Nafion membrane, contact resistance, etc.) (orange text) is calculated by multiplying the HFR (green text) by the current at each potential (blue text, column 4). The iR corrected voltage (column 5) is calculated by adding the V drop to each potential, then plotting it vs the current density to obtain the iR corrected polarization curve (Figure S3D).

References

(1) Moehring, N. K.; Chaturvedi, P.; Cheng, P.; Ko, W.; Li, A.-P. P.; Boutilier, M. S. H. H.; Kidambi, P. R. Kinetic Control of Angstrom-Scale Porosity in 2D Lattices for Direct Scalable Synthesis of Atomically Thin Proton Exchange Membranes. *ACS Nano* **2022**, *16* (10), 16003–16018.

(2) Kidambi, P. R.; Ducati, C.; Dlubak, B.; Gardiner, D.; Weatherup, R. S.; Martin, M.-B. B.; Seneor, P.; Coles, H.; Hofmann, S. The Parameter Space of Graphene Chemical Vapor Deposition on Polycrystalline Cu. *Journal of Physical Chemistry C* **2012**, *116* (42), 22492–22501. https://doi.org/10.1021/jp303597m.

(3) Kidambi, P. R.; Bayer, B. C.; Blume, R.; Wang, Z.-J.; Baehtz, C.; Weatherup, R. S.; Willinger, M.-G.; Schloegl, R.; Hofmann, S. Observing Graphene Grow: Catalyst–Graphene Interactions during Scalable Graphene Growth on Polycrystalline Copper. *Nano Lett* **2013**, *13* (10), 4769–4778. https://doi.org/10.1021/nl4023572.

(4) Cheng, P.; Moehring, N. K.; Idrobo, J. C.; Ivanov, I. N.; Kidambi, P. R. Scalable Synthesis of Nanoporous Atomically Thin Graphene Membranes for Dialysis and Molecular Separations via Facile Isopropanol-Assisted Hot Lamination. *Nanoscale* **2021**, *13* (5), 2825–2837. https://doi.org/10.1039/D0NR07384A.

(5) Kidambi, P. R.; Terry, R. A.; Wang, L.; Boutilier, M. S. H.; Jang, D.; Kong, J.; Karnik, R.; H Boutilier, M. S.; Jang, D.; Kong, J.; Karnik, R. Assessment and Control of the Impermeability of Graphene for Atomically Thin Membranes and Barriers. *Nanoscale* **2017**, *9* (24), 8496–8507. https://doi.org/10.1039/C7NR01921A.

(6) Kidambi, P. R.; Nguyen, G. D.; Zhang, S.; Chen, Q.; Kong, J.; Warner, J.; Li, A.-P. P.; Karnik, R. Facile Fabrication of Large-Area Atomically Thin Membranes by Direct Synthesis of Graphene with Nanoscale Porosity. *Advanced Materials* **2018**, *30* (49), 1–10. https://doi.org/10.1002/adma.201804977.

(7) Cheng, P.; Kelly, M. M.; Moehring, N. K.; Ko, W.; Li, A.-P.; Idrobo, J. C.; Boutilier, M. S. H.; Kidambi, P. R. Facile Size-Selective Defect Sealing in Large-Area Atomically Thin Graphene Membranes for Sub-Nanometer Scale Separations. *Nano Lett* **2020**, *20* (8), 5951–5959.

(8) Chaturvedi, P.; Moehring, N. K.; Cheng, P.; Vlassiouk, I.; Boutilier, M. S. H. H.; Kidambi, P. R. Deconstructing Proton Transport through Atomically Thin Monolayer CVD Graphene Membranes. *J Mater Chem A Mater* **2022**, *10* (37), 19797–19810.

(9) Kidambi, P. R.; Mariappan, D. D.; Dee, N. T.; Vyatskikh, A.; Zhang, S.; Karnik, R.; Hart, A. J. A Scalable Route to Nanoporous Large-Area Atomically Thin Graphene Membranes by Roll-to-Roll Chemical Vapor Deposition and Polymer Support Casting. *ACS Appl Mater Interfaces* **2018**, *10* (12), 10369–10378. https://doi.org/10.1021/acsami.8b00846.

(10) Yoon, S. I.; Seo, D.-J.; Kim, G.; Kim, M.; Jung, C.-Y.; Yoon, Y.-G.; Joo, S. H.; Kim, T.-Y.; Shin, H. S. AA′-Stacked Trilayer Hexagonal Boron Nitride Membrane for Proton Exchange Membrane Fuel Cells. *ACS Nano* **2018**, *12* (11), 10764–10771. https://doi.org/10.1021/acsnano.8b06268.

(11) Inaba, M.; Kinumoto, T.; Kiriake, M.; Umebayashi, R.; Tasaka, A.; Ogumi, Z. Gas Crossover and Membrane Degradation in Polymer Electrolyte Fuel Cells. *Electrochim Acta* **2006**, *51* (26), 5746–5753. https://doi.org/10.1016/J.ELECTACTA.2006.03.008.

(12) Barbir, F. Vehicles with Hydrogen-Air Fuel Cells. In *Energy carriers and conversion systems with emphasis …*; 2009; Vol. II.

(13) Waldrop, K.; Slack, J. J.; Gumeci, C.; Parrondo, J.; Dale, N.; Reeves, K. S.; Cullen, D. A.; More, K. L.; Pintauro, P. N. Electrospun Nanofiber Electrodes for High and Low Humidity PEMFC Operation. *J Electrochem Soc* **2023**, *170* (2), 024507. https://doi.org/10.1149/1945-7111/acb8e2.

(14) Slack, J. J.; Brodt, M.; Cullen, D. A.; Reeves, K. S.; More, K. L.; Pintauro, P. N. Impact of Polyvinylidene Fluoride on Nanofiber Cathode Structure and Durability in Proton Exchange Membrane Fuel Cells. *J Electrochem Soc* **2020**, *167* (5), 054517. https://doi.org/10.1149/1945-7111/ab77fb.

(15) Van Der Linden, F.; Pahon, E.; Morando, S.; Bouquain, D. A Review on the Proton-Exchange Membrane Fuel Cell Break-in Physical Principles, Activation Procedures, and Characterization Methods. *Journal of Power Sources*. 2023, p 233168. https://doi.org/10.1016/j.jpowsour.2023.233168.

(16) Suk, M. E.; Aluru, N. R. Ion Transport in Sub-5-Nm Graphene Nanopores. *J Chem Phys* **2014**, *140* (8), 084707. https://doi.org/10.1063/1.4866643.

(17) Rollings, R. C.; Kuan, A. T.; Golovchenko, J. A. Ion Selectivity of Graphene Nanopores. *Nat Commun* **2016**, *7* (1), 11408. https://doi.org/10.1038/ncomms11408.

(18) W. M. Rohsenow, J. P. Hartnett, E. N. G. *Handbook of Heat Transfer Fundamentals*; 1992; Vol. 1.

(19) O'Hern, S. C.; Stewart, C. A.; Boutilier, M. S. H.; Idrobo, J. C.; Bhaviripudi, S.; Das, S. K.; Kong, J.; Laoui, T.; Atieh, M.; Karnik, R. Selective Molecular Transport through Intrinsic Defects in a Single Layer of CVD Graphene. *ACS Nano* **2012**, *6* (11), 10130–10138. https://doi.org/10.1021/nn303869m.

(20) Fuel Cell Council, U. *USFCC Single Cell Test Protocol # 05-014*; 2006. http://www.members.fchea.org/core/import/PDFs/Technical Resources/MatComp Single Cell Test Protocol 05-014RevB.2 071306.pdf (accessed 2024-07-28).

(21) Balogun, E.; Barnett, A. O.; Holdcroft, S. Cathode Starvation as an Accelerated Conditioning Procedure for Perfluorosulfonic Acid Ionomer Fuel Cells. *Journal of Power Sources Advances* **2020**, *3* (May), 100012. https://doi.org/10.1016/j.powera.2020.100012.

(22) Kabir, S.; Myers, D. J.; Kariuki, N.; Park, J.; Wang, G.; Baker, A.; Macauley, N.; Mukundan, R.; More, K. L.; Neyerlin, K. C. Elucidating the Dynamic Nature of Fuel Cell Electrodes as a Function of Conditioning: An Ex Situ Material Characterization and in Situ Electrochemical Diagnostic Study. *ACS Appl Mater Interfaces* **2019**, *11* (48), 45016–45030. https://doi.org/10.1021/acsami.9b11365.

(23) He, C.; Qi, Z.; Kaufman, A. Electrochemical Method to Improve the Performance of H2/Air Pem Fuel Cells and Direct Methanol Fuel Cells. US6730424B1, 2002. https://patents.google.com/patent/US6730424B1/en (accessed 2024-07-28).

(24) Basha, A. B. M.; Karan, K. Understanding Potential Decay during OCV Hold via Dry Recovery Process. *J Electrochem Soc* **2023**, *170* (6), 064505. https://doi.org/10.1149/1945-7111/acd724.

(25) Takeshita, T.; Kamitaka, Y.; Shinozaki, K.; Kodama, K.; Morimoto, Y. Evaluation of Ionomer Coverage on Pt Catalysts in Polymer Electrolyte Membrane Fuel Cells by CO Stripping Voltammetry and Its Effect on Oxygen Reduction Reaction Activity. *Journal of Electroanalytical Chemistry* **2020**, *871*, 114250. https://doi.org/10.1016/j.jelechem.2020.114250.

(26) Garrick, T. R.; Moylan, T. E.; Carpenter, M. K.; Kongkanand, A. Editors' Choice—Electrochemically Active Surface Area Measurement of Aged Pt Alloy Catalysts in PEM Fuel Cells by CO Stripping. *J Electrochem Soc* **2017**, *164* (2), F55–F59. https://doi.org/10.1149/2.0381702jes.
